# Dynamic evolution and reversibility of single-atom Ni(II) active site in 1T-MoS_2_ electrocatalysts for hydrogen evolution

**DOI:** 10.1038/s41467-020-17904-z

**Published:** 2020-08-17

**Authors:** Brian Pattengale, Yichao Huang, Xingxu Yan, Sizhuo Yang, Sabrina Younan, Wenhui Hu, Zhida Li, Sungsik Lee, Xiaoqing Pan, Jing Gu, Jier Huang

**Affiliations:** 1 Department of Chemistry, Marquette University, Milwaukee, WI 53201 USA; 2 Department of Chemistry and Biochemistry, San Diego State University, San Diego, CA 92181 USA; 3 Key Lab of Organic Optoelectronics & Molecular Engineering of Ministry of Education, Department of Chemistry, Tsinghua University, 100084 Beijing, PR China; 4 Department of Materials Science and Engineering, University of California, Irvine, CA 92697 USA; 5 X-ray Science Division, Argonne National Laboratory, Argonne, IL 60349 USA; 6 Department of Physics and Astronomy, University of California, Irvine, CA 92697 USA

**Keywords:** Catalytic mechanisms, Energy, Electrocatalysis, Nanoscale materials

## Abstract

1T-MoS_2_ and single-atom modified analogues represent a highly promising class of low-cost catalysts for hydrogen evolution reaction (HER). However, the role of single atoms, either as active species or promoters, remains vague despite its essentiality toward more efficient HER. In this work, we report the unambiguous identification of Ni single atom as key active sites in the basal plane of 1T-MoS_2_ (Ni@1T-MoS_2_) that result in efficient HER performance. The intermediate structure of this Ni active site under catalytic conditions was captured by in situ X-ray absorption spectroscopy, where a reversible metallic Ni species (Ni^0^) is observed in alkaline conditions whereas Ni remains in its local structure under acidic conditions. These insights provide crucial mechanistic understanding of Ni@1T-MoS_2_ HER electrocatalysts and suggest that the understanding gained from such in situ studies is necessary toward the development of highly efficient single-atom decorated 1T-MoS_2_ electrocatalysts.

## Abstract (web summary)

While single atom catalysis combines heterogeneous materials with molecular understanding, the role of the single atoms remains vague. Here, authors examine single Ni on MoS_2_ via in situ X-ray absorption spectroscopy to reveal the intermediate and catalytically active species.

## Introduction

As the world continues to transition toward the usage of carbon neutral energy technologies, there remains a need to develop efficient and cost-effective hydrogen evolution reaction (HER) catalysts to support the development of a hydrogen economy^1,2^. A very promising HER catalyst that meets both the technical and economical requirements is MoS_2_^3–5^. Tremendous efforts have been made to clarify the catalytic mechanism with various morphological and compositional MoS_2_, such as chemical exfoliated MoS_2_^6,7^, nanostructured particles^4,8–11^, heterostructures^11,12^, amorphous^13–17^, and doped MoS_2_^18–20^ etc. Both experimental and computational studies demonstrated that the edge sites of the crystalline MoS_2_ are catalytically active, while its basal plane is inert^3,5,21^. As a result, one desirable approach to further improving the activity of MoS_2_ is to activate the inert basal plane.

1T-MoS_2_, which features a well-defined octahedral symmetry in contrast to the traditional trigonal prismatic 2H-phase MoS_2_^22,23^, represents an emerging platform for further improving the HER performance of MoS_2_ owing to their great potential in activating basal planes. This is not only theoretically predicted that the metallic 1T-MoS_2_ may have basal plane active sites^24,25^ but also justified by recent experiment where MoS_2_ tethered with single-atom catalyst at basal plane can effectively enhance the catalytic activity of 1T-MoS_2_^26–28^_._ However, whether the single atoms in the basal edge act as active sites and what the exact function of these single atoms in 1T-MoS_2_ remain unclear. It is thus essential to seek direct evidence for the origins of active sites due to the single-atom modification from an experimental aspect of view.

In this work, we report the direct observation of single-atom Ni replacing Mo and S as active sites on basal edge of the Ni@1T-MoS_2_ HER electrocatalyst in the acidic condition. The Mo and Ni atoms both adopt octahedral structure in the 1T phase of MoS_2_. Using in situ X-ray absorption spectroscopy (XAS), we show that the dominant active site for HER is the Ni single atom in its intrinsic environment in an acidic electrolyte, while, in alkaline media, Ni single atoms reconstruct into an S-supported NiO species and reversibly forms a metallic active species under applied potential. These findings provide important insight into the dynamic evolution of basal plane Ni active sites in a state-of-the-art 1T-MoS_2_ HER electrocatalyst, thereby revealing key intermediate and active species that dictate the catalytic function.

## Results

### Synthesis, characterization, and electrocatalysis

**Fig. 1:**
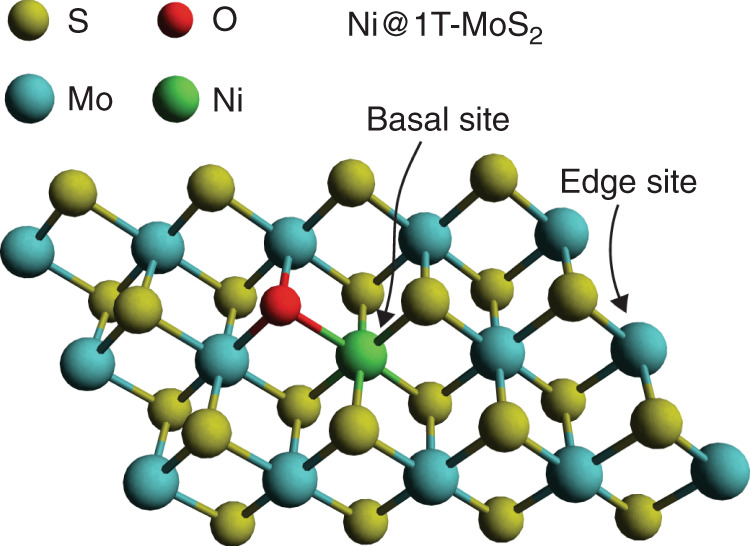
Ni@1T-MoS_2_ structure. Segment of 1T-MoS_2_ structure with basal plane dopant NiO moiety shown in the center.

The synthesis of Ni@1T-MoS_2_ HER electrocatalyst follows the previously published protocols^28^. The synthesis procedure was conducted as follows. First, a Anderson-type POM nanocluster precursors, [(NH_4_)_4_[NiH_6_Mo_6_O_24_]·5H_2_O (NiMo_6_) were prepared. It contains well-defined, 1:6 heteropolyanion clusters, composed of a single metal heteroatom NiO_6_ octahedron with six edge-sharing MoO_6_ octahedrons. The as-prepared NiMo_6_ precursors react with thioacetamide at 180 °C for 24 h in the presence of carbon fiber paper (CFP, 1 × 2 cm^2^) to give rise to the corresponding Ni@1T-MoS_2_/CFP electrocatalyst. The loading amount of Ni@1T-MoS_2_ on CFP is about 1 mg cm^−2^. The facile sulfuration reaction incompletely replaces O atoms with S atoms and generates 1T-MoS_2_ nanosheets randomly decorated with Ni single atoms (Fig. 1)^28^.

**Fig. 2:**
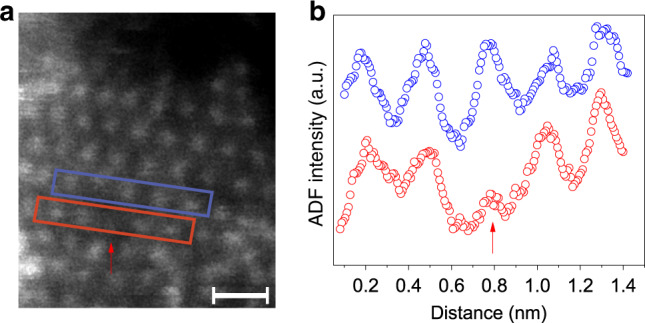
STEM characterization of Ni@1T-MoS_2_ sample. **a** Atomic resolution ADF-STEM image of monolayer sample at 60 keV. The length of the scale bar is 5 Å. **b** Intensity profiles taken along two adjacent lines indicated by blue and red rectangles in (**a**). The red arrow points to the location of Ni single atom.

The as-prepared Ni@1T-MoS_2_ catalyst was extensively characterized by scanning electron microscopy (SEM), transmission electron microscopy (TEM), energy dispersive X-rays spectroscopy (EDX), Raman spectrum, and powder X-ray diffraction (XRD) (Supplementary Fig. 1). SEM image shows that ultrathin Ni@MoS_2_ coated evenly throughout the CFP (Supplementary Fig. 1a). More detailed structures of Ni@MoS_2_ were further demonstrated by TEM images (Supplementary Fig. 1b). The XRD patterns of Ni@1T-MoS_2_ electrocatalyst show clear characteristic diffraction peaks at around 10.35°, 32.84°, and 35.70° (red plot in Supplementary Fig. 1c), which correspond to the (002), (100), and (102) planes of hexagonal MoS_2_ (PDF#75-1539), respectively. However, the peak of Ni@1T-MoS_2_ at 2θ = 10.25° represents a stacked, multilayered structure, which is lower than that of MoS_2_ (PDF#75-1539) at 14.13°, indicating that this multilayered spacing of 1T-MoS_2_ is larger than that of the bulk 2H-MoS_2_ (6.3 Å, PDF#75-1539). Previous publications also verified analogous XRD patterns for 1T-MoS_2_ or few-layered 2H-MoS_2_^7,27^. The Raman peaks of Ni@1T-MoS_2_ resemble that of 1T-MoS_2_, which is consistent with their similar structures. The characteristic Raman peaks at 147, 214, 236, 283, and 335 cm^−1^ (Supplementary Fig. 1f) can be assigned to the phonon modes in 1T-MoS_2_, which suggests the formation of a pure 1T-MoS_2_ nanosheet. These results together confirmed the phase of MoS_2_ to be 1T, similar to previous reported 1T-MoS_2_ work (Supplementary Fig. 1c, f)^28–30^. Furthermore, the Ni@1T-MoS_2_ showed similar structure as pristine 1T-MoS_2_, indicating that the addition of Ni did not change the structure of 1T-MoS_2_. EDX analysis confirms that Mo, Ni, S, and O atoms are uniformly distributed in Ni@1T-MoS_2_ electrocatalyst (Supplementary Fig. 1d, e). The uniform distribution of oxygen (O) element indicates that sulfur atom (S) only partially replaced O in the POM precursor, which is similar to the previous study^7,28,30^. Furthermore, STEM (scanning TEM) confirmed that the single-layer 1T-MoS_2_ consists of symmetric hexagon units, which corresponds to the 1T-MoS_2_ structure with intensity variation, i.e., Ni atoms are shown as darker dots and Mo atoms are shown as brighter dots (Fig. 2a). In the annular dark-field (ADF) STEM images, the intensity of ADF signal is proportional to the atomic number (*Z*) of observed materials and scales as *Z*^1.6–2^^31^. Therefore, the STEM intensity of single doped Ni atom (*Z*_Ni_ = 28) should be dimmer than that of Mo atom (*Z*_Mo_ = 42) in MoS_2_ lattice. The simulated spectrum intensity matches with the experimental intensity, indicating that the bright spots are Mo and dark spots are Ni, rather than a vacant site in STEM images (Fig. 2b). Different from most single-atom modified MoS_2_, where heavier single atoms are usually shown as brighter spots on the substrate^32^, here the intensity variation confirms that Ni has successfully replaced Mo. In addition, the HAADF experimental and simulated intensity profiles further show that the dark and bright spots are corresponding to Ni sites and Mo sites (Supplementary Fig. 2), respectively.

The catalytic activity of the synthesized Ni@1T-MoS_2_ was examined by linear sweep voltammetry (Supplementary Fig. 3), which shows significantly enhanced catalytic activity in Ni@1T-MoS_2_ compared to pristine 1T-MoS_2_ in both acidic and alkaline electrolyte, with a more prominent difference in alkaline electrolyte. Ni@1T-MoS_2_ showed ~80 mV more positive onset potential in 0.5 M H_2_SO_4_ while its onset potential shifted ~300 mV catholically in 1M NaOH. The HER stability of the as-prepared Ni@1T-MoS_2_ in terms of chronoamperometry was also examined, which exhibited excellent stability in both acidic and basic conditions at the applied −0.76 V vs. RHE, which is the potential later used for the in situ XAS analysis (Supplementary Fig. 4).

### In situ XAS under acidic and alkaline conditions

In situ XAS was used to directly measure the local bonding and electronic structures at Ni and Mo centers under the standard catalytic conditions in both acidic and alkaline media. In both acidic and alkaline media, 0 V vs. RHE represents a pre-catalytic potential and −0.76 V vs. RHE represents a proceeding catalytic potential for HER. All potentials reported in this work are with respect to RHE if not indicated. In addition to these potentials, samples were measured as-prepared (dry sample) and with no applied potential, representing a sample that is immersed in electrolyte with N_2_ purging but has not been externally connected to a potentiostat.

**Fig. 3:**
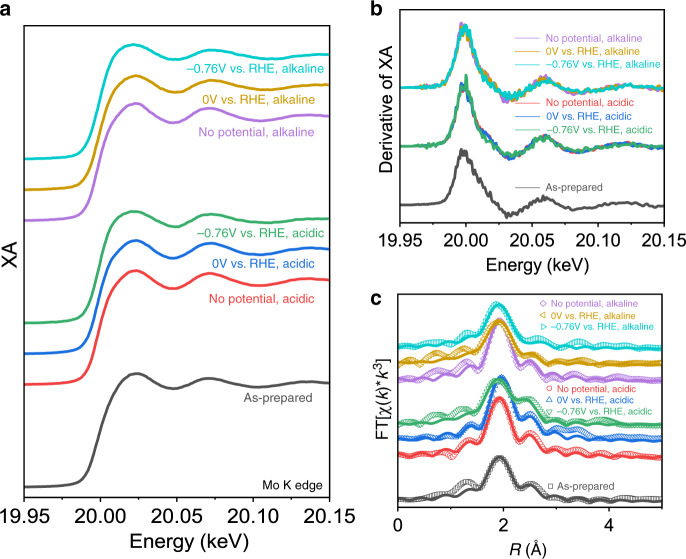
In situ XAS at Mo K-edge. **a** XANES spectra, **b** first-derivative spectra, and **c** Fourier-transformed R-space spectra (open points) and fits (solid lines) of Ni@1T-MoS_2_.

Figure 3a shows the X-ray absorption near-edge structure (XANES) region of the XAS spectrum collected at each condition at the Mo K-edge. In both acidic and alkaline media, the XANES spectra at Mo K-edge, corresponding to the 1s–5p transition, show very little change in either shape or edge position as shown by the first-derivative spectra (Fig. 3b) as a function of applied potential, suggesting negligible structure or oxidation state changes. The extended X-ray absorption fine structure (EXAFS) spectra (Supplementary Fig. 5) are plotted in R-space in Fig. 3c.

The EXAFS data were analyzed using FEFF fitting via the Demeter suite of programs^33^ to extract quantitative Mo local structure information. The published 1T-MoS_2_ crystal structure was used to build FEFF models^34^. The EXAFS spectra in R-space and k-space, as well as the fitting results, are presented in Fig. 3c and Supplementary Fig. 6, respectively, where their corresponding fitting parameters are listed in Supplementary Table 1. Further explanation of the model is given in the Experimental section and Supplementary Fig. 7. Two relatively minor changes are observed at the Mo K-edge via in situ XAS. The first is an increase in coordination number (CN) with little change in the correlated Debye–Waller factor (σ^2^) after immersion in electrolyte, implying solvent coordination to undercoordinated Mo centers. The second is the shortening of Mo–S distance under catalytic applied potential (i.e., −0.76 V) in both acidic and alkaline conditions. Beyond this slight change, the Mo centers under applied catalytic potential appear to have largely similar local structure to the as-prepared sample and the small changes observed are insufficient for us to draw decisive conclusions. This result is different from a previous report on amorphous MoS_x_, where significant structural changes at Mo center were observed^13^. It is unsurprising, however, as Ni in this work was shown to be the active catalyst site in Ni@1T-MoS_2_. One additional activation mechanism that needs to be considered is lattice structure changes, namely a phase change from the 1T phase to the 2H phase. Based on our previous work^28^, and published work^35–37^, the 2H phase gives a clear second-shell Mo–Mo peak while 1T-MoS_2_ does not have a clear second-shell peak. Because no such feature is observed under any of the measured conditions in situ, we conclude that the lattice does not undergo a full transition to the 2H phase and can also rule out a 1T-2H polymorphic state as the catalytically active state. Therefore, the 1T phase is shown to be the catalytically active phase in both acidic and alkaline conditions with minimal changes at the Mo center and discussion hereafter focuses on the major changes observed at the Ni K edge.

**Fig. 4:**
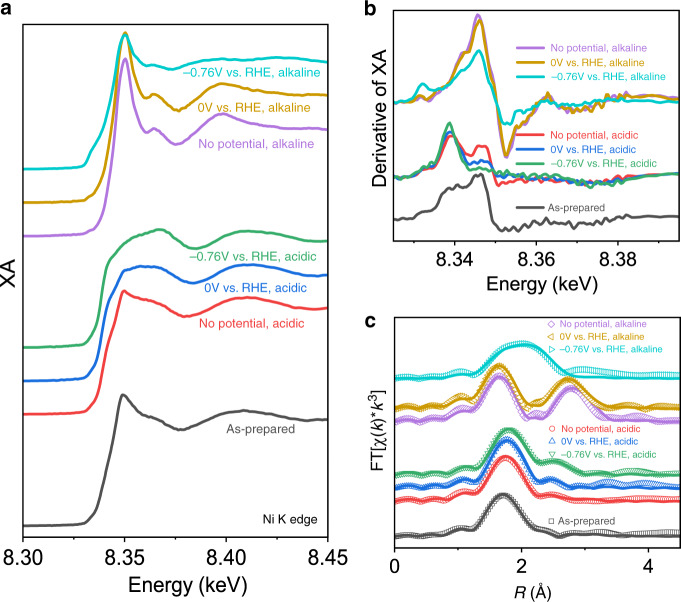
In situ XAS at Ni K-edge. **a** XANES spectra, **b** first-derivative spectra, and **c** Fourier-transformed R-space spectra (open points) and fits (solid lines) of Ni@1T-MoS_2_.

The Ni–K edge EXAFS spectra (Supplementary Fig. 5b) were measured under the same conditions as Mo K-edge data. The XANES spectra (Fig. 4a) show that significant local structure changes occur throughout the catalysis process. Beginning from the dry sample, a weak white line feature is observed on-edge (1s–4p transition, 8.350 keV). Such a feature is typically observed for Ni^2+^ with unsaturated coordination and is sensitive to the identity of coordinating atom^38–41^, suggesting that single-atom Ni preserves an oxidation state of 2+ after replacing Mo atoms. The oxidation state is further supported by Ni reference spectra (Supplementary Fig. 8). Moreover, no peak is observed at 2.10 Å in the R-space EXAFS spectra (Fig. 4c) where Ni–Ni coordination (first shell) is, correlating well with the previous conclusion that Ni exists as single atom in 1T-MoS_2_. Upon immersing in acidic electrolyte (No potential), it is observed that the shape of the spectrum at above-edge region has little change; however, a shoulder (~8.340 keV) is observed on the rising part of the edge along with a reduced white line intensity. When the first-derivative spectra are compared (Fig. 4b), it is apparent that the edge shifts by approximately 3 eV to a lower energy. While this is consistent with reduction of the Ni metal center without applied potential^42–44^, structural and electronic factors could contribute and are examined in more detail via EXAFS fitting (*vide infra*). Further shifting of the edge toward lower energies is observed with the application of pre-catalytic (0 V) and catalytic (−0.76 V) potentials.

Immersion in alkaline electrolyte yields strikingly different results compared to the acidic electrolyte. Without applied potential, a strengthening in the intensity of the 1s–4p white line transition is observed (Fig. 4a), which occurs without an apparent change in oxidation state (Fig. 4b). The XANES spectrum for no potential, alkaline appears to resemble NiO (Supplementary Fig. 9a, b), however the local structure revealed by EXAFS suggests a very different species (Supplementary Fig. 9c, d) still associated with 1T-MoS_2_. The application of a pre-catalytic potential (0 V) results in little change in the spectrum and yields no change in edge position. Applying a catalytic potential (−0.76 V) causes a significant change in the XANES spectrum. A shoulder at the base of the spectrum (8.334 keV) appears to grow while above-edge oscillations >8.350 keV are changed and dampened. The first-derivative spectrum (Fig. 4b) shows that a new edge feature consistent with the position of the shoulder is observed at 8.333 keV. Evidently, this peak is lower in energy than the species observed in acidic media and is consistent with the formation of a Ni^0^ species, as the Ni metal edge position is 8.333 keV^45^.

The Fourier-transformed R-space spectra (Fig. 4c) were fit to further extract quantitative information about species formed at the Ni center. The K-space spectra and fits are shown in Supplementary Fig. 10. Beginning with acidic conditions, no significant changes are observed in the quantified local structure FEFF parameters (Table 1) as a function of applied potential in the first coordination shell. However, a shortening of the Ni–Mo second-shell interaction is observed upon immersion in acidic electrolyte and further shortening due to applied potential. Compared to the Mo K edge results, this result does appear to be consistent with the shortening of first shell Mo–S first shell interactions observed under an applied bias of −0.76 V. At the no potential, acidic and 0 V vs. RHE conditions, where changes were not observed at the Mo K-edge, a shortening of Ni–Mo in the first shell is still observed. This is likely correlated with the oxidation state changes observed only at Ni that result in local structure changes, whereas the Mo K-edge probes an average of all Mo atoms irrespective of their proximity to Ni sites. Overall, however, these results suggest that the intrinsic Ni site in Ni@1T-MoS_2_ is catalytically active and does not undergo significant transformation into a different active species in acidic conditions.

**Table 1:** FEFF fitting parameters for Ni–K edge in situ XAS data.

Condition	Vector	CN^a^	σ^2^ (Å)^2b^	Δ*E*_0_ (eV)	*R* (Å)^c^
As-prepared	Ni–S	3.6	0.006	−8.01	2.20
	Ni–O	1.0	0.006	−1.03	2.37
	Ni–Mo	0.8	0.010	−15.44	3.10
No potential, acidic	Ni–S	3.5	0.007	−5.52	2.21
	Ni–O	1.0	0.007	−5.48	2.41
	Ni–Mo	1.3	0.010	−13.22	3.02
0 V vs. RHE, acidic	Ni–S	3.6	0.006	−2.52	2.21
	Ni–O	1.0	0.006	−1.39	2.37
	Ni–Mo	1.0	0.012	−16.37	2.96
−0.76 V vs. RHE, acidic	Ni–S	3.6	0.006	0.35	2.22
	Ni–O	1.0	0.006	6.53	2.38
	Ni–Mo	2.8	0.010	−20.75	2.97
No potential, alkaline	Ni–S	1.7	0.010	0.58	2.21
	Ni–O	4.2	0.006	0.58	2.05
	Ni–Ni	4.9	0.008	6.13	3.12
0 V vs. RHE, alkaline	Ni–S	1.9	0.008	−0.25	2.20
	Ni–O	3.8	0.007	−0.25	2.04
	Ni–Ni	3.9	0.008	1.26	3.08
−0.76 V vs. RHE, alkaline	Ni–S	1.0	0.001	4.65	2.20
	Ni–O	2.1	0.009	−0.79	2.07
	Ni–Ni	2.1	0.005	5.53	2.53

^a^Coordination number, uncertainty ± 0.5.

^b^Uncertainty ± 0.001 Å^2^.

^c^Uncertainty ± 0.02 Å.

The R-space spectra (Fig. 4c) under alkaline conditions, however, suggest that the active species is not the intrinsic Ni species in as-prepared Ni@1T-MoS_2_. Upon immersion in alkaline electrolyte, a significantly different second-shell scattering feature is observed, which is accompanied by an obvious shortening in the first-shell peak distance. In the first shell, such a shortening is consistent with enhanced Ni–O coordination and reduced Ni–S coordination. As discussed in the XANES results, the local structure is not consistent with a pure NiO phase but rather a phase still associated with 1T-MoS_2_. The FEFF model therefore incorporated Ni–O first shell and Ni–S first shell scattering, where it was observed that Ni is predominantly coordinated to O with a smaller contribution of S compared to the as-prepared dry sample. As this species is not NiO, we term the species NiS_x_O_y_, where *x* and *y* represent the CNs of their respectively labeled atoms, as suggested by EXAFS analysis (Table 1) and not the stoichiometric amounts of the atoms in the entire catalyst material. The newly observed second-shell feature is intense relative to the first shell peak and indicates the presence of a large scattering atom in the second shell of Ni. In Ni@1T-MoS_2_, such a scattering atom could be either Ni or Mo. If the second-shell scattering was Ni–Mo, then second-shell scattering should be also observed from the Mo K-edge. Because no significant second-shell scattering was observed under identical conditions in the Mo K edge data (Fig. 3c), it is more likely due to Ni–Ni second-shell scattering. Evidently, alkaline conditions even in the absence of applied potential impart lability to Ni and leads to the formation of Ni–O–Ni moieties in the structure. Based on the FEFF fit results in Table 1, the Ni–O–Ni moieties are still anchored by direct coordination to S atoms within the 1T-MoS_2_ structure, i.e., an NiS_x_O_y_ structure.

Application of a pre-catalytic potential (0 V) does not significantly change the Ni local structure compared to the no potential alkaline electrolyte measurement. When a catalytic potential (−0.76 V) is applied in alkaline electrolyte, the R-space spectrum becomes to be a very broad feature spanning the entire first-shell region of the spectrum (Fig. 4c). Combined with the observation of Ni^0^ species in the XANES spectra, we assigned the new long-distance first shell feature to the formation of Ni–Ni coordination concurrent with reduction of Ni to Ni^0^. We can exclude the formation of Ni–Mo bonds because no changes were observed at the Mo K-edge under the same conditions. The optimum FEFF model therefore incorporated Ni–S, Ni–O, and Ni–Ni paths, giving a species that is partly metallic in nature and is coordinated to the 1T-MoS_2_ scaffold via O and S bonds. To confirm the validity of this claim, we performed FEFF fitting of Ni foil (Supplementary Fig. 11 and Supplementary Table 2) where it was observed that the fit Ni–Ni distance matches the catalytic alkaline condition within uncertainty.

### Dynamic evolution and reversibility of Ni species

**Fig. 5:**
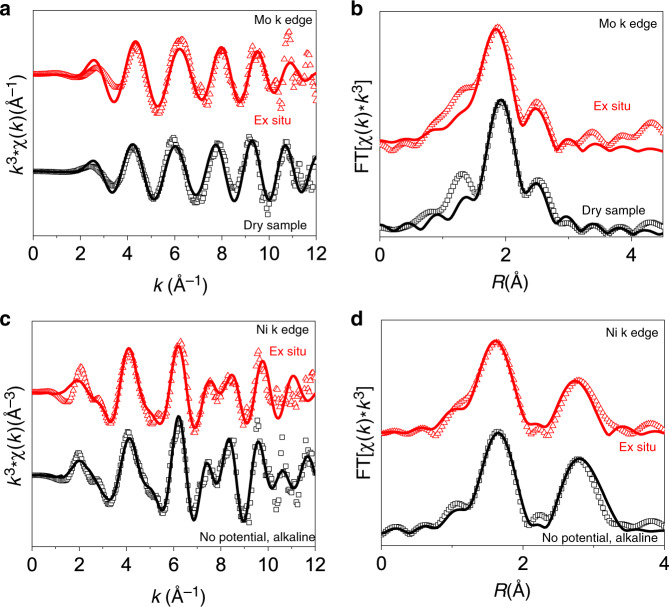
Ex situ XAS after alkaline electrocatalysis. **a** Mo K edge K-space spectra and **b** R-space spectra and **c** Ni K edge K-space spectra and **d** R-space spectra ex situ EXAFS results, which are compared to the applicable results in this work.

Given the nature of the active site in alkaline conditions, we performed post-catalysis ex situ XAS on Mo K-edge and Ni K-edge Mo K-edge (Supplementary Fig. 12) and Ni K-edge (Fig. 5). The Mo K-edge shows a slight shortening in Mo–S distance, however, the FEFF model adequately describes the local structure owing to the stability of the catalyst. The Ni K-edge gives a Fourier-transformed R-space spectrum very similar to the no potential and 0 V spectra in alkaline conditions, i.e., the NiS_x_O_y_ structure. Indeed, FEFF fitting parameters confirm that these species are indeed the same, within uncertainty. In full, this implies that the metallic Ni phase that forms as a result of catalytic potentials is indeed reversible, i.e., Ni returns to its original oxidation state and local structure rather than remaining in a metallic phase under applied bias. The insights gained in this study are limited by the inability to directly observe atom migration in situ to explain the migration of Ni atoms that are initially well-dispersed in the structure without Ni neighbors^28^. However, surface reconstruction in 2D materials^13,46,47^ and other materials^48^ has been reported before. Relatedly, defect and vacancy sites have been shown to be important in MoS_2_-catalyzed HER^49,50^. Furthermore, electrolyte dependent performance and electrolyte-induced dopant local structure changes have been observed in MoS_2_^51,52^. We therefore posit that alkalinity induces Ni atom lability within the basal plane allowing the formation of Ni–O–Ni moieties, and leads to the formation of Ni^0^ under applied catalytic potential.

**Fig. 6:**
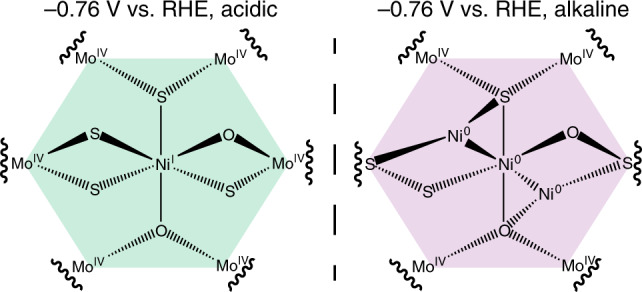
The proposed Ni@1T-MoS_2_ active species. The active species under acidic (left) and alkaline (right) conditions. Under alkaline conditions, the structure shown is representative, and the Ni^0^ cluster size is further discussed in the text.

The active species as suggested by this work are depicted in Fig. 6. In both acidic and alkaline electrolyte, the Mo K-edge results indicate that the 1T phase is maintained and harbours the active Ni species that was investigated by probing the Ni K edge under identical conditions. Under acidic conditions, Ni was shown to remain coordinated by predominantly S atoms and underwent reduction under applied catalytic potential. In alkaline conditions, the active species was determined to be Ni^0^ in nature and Ni–Ni coordination was observed directly via EXAFS. In addition to Ni–Ni coordination, it was observed that Ni–O and Ni–S coordination was still present, suggesting that the active species is still anchored within the basal plane of 1T-MoS_2_.

The CN of Ni–Ni in the alkaline active species was found to be ~2. If we consider that Ni^0^ atoms form a cluster within the 2D 1T-MoS_2_ sheet that is bound on its periphery by the lattice, an average CN of 2 suggests that the size of Ni^0^ clusters is small such that some Ni atoms contain >2 neighbors while others contain <2 neighbors. We therefore estimate that the cluster size is on the order of 10 Ni atoms or less. Furthermore, clear Ni–Ni second-shell interactions were not observed in the data for the catalytically active species, suggesting that a pure Ni metal phase is not observed. The lattice-contained small Ni cluster proposed here, however, supports the lack of a second-shell Ni–Ni feature, as a small, disordered cluster would inherently not give such a feature due to disorder.

Further, from the comparison in pre- and post-electrolysis XPS spectra and ICP-MS solution analysis, we have found that Ni@MoS_2_ is quite stable under acidic conditions, correlating well with the XAS results. To further confirm that Mo does not go to Mo (0), as shown in Supplementary Fig. 13, the high-resolution (HR) Mo 3d spectra were mainly deconvoluted into two peaks in the original and acid-operated samples. These two characteristic peaks of Mo 3d_5/2_ (~228.5 eV) and Mo 3d_3/2_ (231.6 eV) suggest the dominating oxidation state of Mo is +4 in Ni@1-TMoS_2_)^53^. Interestingly, the Mo 3d peaks in basic condition shows a slight shift of ~0.3 eV to higher binding energy compared to those of original and acidity ones. Moreover, a higher Mo^6+^ peak is generated after electrolysis under basic condition, indicating that Ni@1T-MoS_2_ catalyst probably undergoes a surface oxidation in the electrolysis process. Combining XAS and XPS results, we believe that Mo does not go to Mo (0) in HER. However, the catalysts are oxidized in alkaline conditions, reflected by the increased binding energies in Mo3d, Ni 2p, and S 2p peaks (Supplementary Fig. 13). This is consistent with the XAS result that Ni–O coordination increases in Ni@1T-MoS when exposed to alkaline electrolyte. Further ex situ characterization was performed via Raman spectroscopy. Raman spectra commonly detect at a much deeper material depth (μm range) than XPS (several nm), and are therefore more sensitive to the bulk structure of the catalyst. The Raman spectra (Supplementary Fig. 14) indicate that the bulk material is stable after 30 h of electrolysis. This is in good agreement with EXAFS results, where a 1T to 2H phase transition is not observed, supporting that the active catalyst remains in the 1T phase. ICP-MS was further applied to detect leaching into the electrolyte after electrolysis (Supplementary Table 3). In acidic conditions, no detectable leaching was observed. In alkaline conditions, no leaching of the catalytically active Ni species was observed, and 0.47 µg mL^−1^ Mo was observed, suggesting that, as further suggested by XPS (Supplementary Fig. 13), oxidized Mo species gradually leach during long catalysis runs in alkaline electrolyte. We note that these leached Mo species were not observed in EXAFS measurements likely due to their low concentration in solution relative to the Mo concentration on the electrode.

Because of the observed reversibility, the second-shell Ni–Ni scattering in the alkaline sample without applied potential should also be informative toward the size of Ni–O clusters in the pre-catalytic states. A fit CN of ~4 was obtained in both the in situ (Table 1) and ex situ (Supplementary Table 4) data for the second-shell Ni–Ni path. This implies that all Ni atoms in second-shell proximity do not become associated in the first shell under applied catalytic potential. This is likely due to the mixed coordination with the 1T-MoS_2_ lattice as observed in the alkaline −0.76 V in situ XAS data. This result is significant since it clarifies the central role of single-atom Ni, replacing Mo and S, as the main active species rather than as promoters in both acidic and alkaline condition. Moreover, under alkaline condition, single-atom Ni undergoes a structure evolution from forming Ni–O, followed by Ni–Ni species. Interestingly, without bias, the reduced species possibly can be regenerated by oxidation from oxygen and return back to Ni–O clusters.

In summary, we performed in situ XAS to investigate the active site structure of the high-performance Ni@1T-MoS_2_ HER electrocatalyst at both Mo and Ni k-edge. It was discovered that Mo sites undergo very little change during the process of immersion into electrolyte and consequent potential-driven electrocatalysis. At the active Ni single-atom sites, however, significant changes are observed. In acidic electrolyte, the active site structure of Ni center experiences a net reduction in oxidation state without change of intrinsic structure during catalysis. In contrast, under alkaline conditions, coordination changes are observed from immersion in electrolyte, where NiS_x_O_y_ species form, and the application of a catalytic potential reversibly forms a metallic Ni species as the active site. These findings provided direct evidence that single-atom Ni(II) can itself perform as active species at the interface of Ni@1T-MoS_2_ in acidic conditions while it undergoes structural reconstruction in the alkaline medium to form a NiS_x_O_y_ species that reversibly forms a catalytically active Ni^0^ species under applied potential. Moreover, since most Ni single atoms are placed on the basal edge of 1T-MoS_2_, these results also support the fact that Ni sites in the basal plane of Ni@1T-MoS_2_ are active towards HER under acidic conditions and sheds light on the function and evolution of Ni sites in 1T-MoS_2_ under alkaline conditions.

## Methods

### Preparation of NiMo_6_ precursor

All chemicals were obtained as reagent grade chemicals from Alfa Aesar^®^ unless noted. The (NH_4_)_4_[NiH_6_Mo_6_O_24_]·5H_2_O (NiMo_6_) precursor was prepared according to a modified published procedure. (NH_4_)_6_Mo_7_O_24_·4H_2_O (denoted Mo7, 5.19 g, 4.2 mmol, 99%) was dissolved in DI water (80 mL) and then heated to 100 °C. Ni(NO_3_)_2_·6H_2_O (1.16 g, 4 mmol, 99.99%) was dissolved in water (20 mL), which was added to the above solution with stirring. The mixture was kept heating and stirring to give rise to a deep green solution. The crude product (5.4 g) was isolated with evaporation and filteration. The green targeted product (4.6 g, 79.1% yield based on Mo) was obtained by recrystallization in hot water (80 °C) two times, then dried under vacuum. Elemental analysis calcd (%) for H32N4O29NiMo6 (M = 1186.60 gmol^−1^): H, 2.72; N, 4.72; Mo, 48.51; Found: H, 2.70; N, 4.66; Mo, 48.62. IR (KBr pellet, major absorbances, cm^−1^): 3402 (ν_as_OH, m), 3152 (ν_as_NH, m), 1627 (δOH, m), 1402 (δNH, s), 929 (νMo = O, vs), 876 (νMo = O, vs), 635 (νMo–O–Mo, vs), 577 (νNi–O–Mo, w).

### Preparation of Ni@1T-MoS_2_/CFP

The Ni@1T-MoS_2_ catalyst was prepared according to our previous work^28^. The as-prepared NiMo6 (50 mg, 0.042 mmol) precursors, thioacetamide (TAA, 80 mg, 1.065 mmol, 98%) and CFP (1 × 2 cm^2^, Toray carbon paper, TGP-H-60) were mixed in 10 mL H_2_O, transferred into a 20 mL Teflon autoclave, and heated at 180 °C for 24 h to give rise to the corresponding Ni@1T-MoS_2_/CFP electrocatalyst. The loading amount of Ni@1T-MoS_2_ on CFP is about 1 mg cm^−2^. (Note: CFP represents conductive CFP, which can serve as substrate to enable the loading of Ni@1T-MoS_2_ catalyst, forming Ni@1T-MoS_2_/CFP).

### Preparation of 1T-MoS_2_

80 mg thioacetamide and 50 mg (NH_4_)_6_Mo_7_O_24_·4H_2_O (Alfa Aesar) were dissolved in 10 mL DI water under sonication treatment for 20 min to form homogeneous solution. Afterwards the solution was transferred into a 25 mL Teflon autoclave, and CFP (1 × 2 cm^2^) was placed onto the bottom. The Teflon autoclave was heated at 180 °C for 24 h to give rise to the in situ growth of 1T-MoS_2_ on CFP substrate. In addition to the 1T-MoS_2_ grown on the CFP, the remained free 1T-MoS_2_ was collected by centrifugation and then was washed with DI water, ethanol and acetone (each for two times). The purified 1T-MoS_2_ was dried in thermal oven for overnight and ground into fine powders. Loading amount of 1T-MoS_2_ on CFP is quantified by 0.5 mg/cm^2^ by comparing the mass difference before and after hydrothermal growth.

### Material characterization

The morphology and size of the nanostructured materials were characterized by a HITACHI H-7700 TEM with an accelerating voltage of 100 kV, and a FEI Tecnai G2 F20 S-Twin HR TEM, operating at 200 kV on a HITACHI S-5500. Aberration-corrected STEM imaging was performed by Nion UltraSTEM 200 at UC-Irvine, equipped with C3/C5 corrector and high-energy resolution monochromated EELS system (HERMES). The instrument was operated at accelerating voltage of 60 kV with convergence semi-angle of 38 mrad and with a beam current of ~10 pA to reach atomic resolution. For STEM imaging, the inner and outer collection semi-angles of ADF detector were 70 and 210 mrad respectively. SEM with energy dispersive X-ray spectroscopy equipment was conducted on a LEO 1530. Raman spectra were recorded using a HORIBA JY HR800 confocal Raman microscope, employing an Ar-ion laser operating at 532 nm. The Raman spectra of Ni@1T-MoS_2_ show distinct peaks from 2H-MoS_2_ (379, 404, and 454 cm^−1^)^54^, as well as the new peaks between 100 and 375 cm^−1^ (i.e., 147, 198, 240, 283, and 345 cm^−1^), corresponding to the phonon modes in 1T-MoS_2_. Powder XRD characterization was performed on a Bruker D8 Advance X-ray diffractometer using Cu-Kα radiation (*λ* = 1.5418 Å).

### In situ XAS measurements

In situ XAS was performed at beamline 12-BM at the Advanced Photon Source, Argonne National Laboratory. Fluorescence mode detection was used for all samples using a 13-element germanium solid state detector (Canberra). Reference metal foil spectra were collected in transmission mode with ion chambers for energy calibration.

The dry sample spectra were collected in air, while all other samples were collected in a customized 3-electrode in situ electrochemical cell (Supplementary Fig. 15). For the acidic condition, 0.5M H_2_SO_4_ was used as the electrolyte whereas the alkaline condition used 1M KOH. The electrode is positioned in front of the Kapton window to give the minimum possible path length through electrolyte. The cell was purged with N_2_ for 15 min before beginning experiments. The cell contains four inlets, one for working electrode clamp which was connected to the sample on CFP, one reference electrode (SCE), and a graphite rod counter electrode. The other inlet is a small hole for a Teflon tubing for purging. Finally, another small hole acts as the purge outlet to exhaust purge gas as well as gas produced during electrocatalysis reactions.

Potentials were applied during in situ experiments using an EC Epsilon potentiostat and controlled potential electrolysis experiment. Prior to applying the 0 V potential, a linear sweep voltammetry experiment was performed to confirm function of the catalyst. The applied potentials were determined using the PH value of 1 for acidic condition and PH value of 14 for the alkaline condition. The correct potential applied during in situ experiments was calculated against the SCE reference via Eq. 1, using a value of +0.241 V vs. RHE for E^0^SCE:

1 $${\mathrm{E}}\left( {{\mathrm{RHE}}} \right) \,=\, {\mathrm{E}}\left( {{\mathrm{SCE}}} \right) + 0.059 \, {\mathrm{pH}} \,+\, {\mathrm{E}}^0{\mathrm{SCE}}.$$

### XAS data analysis

XAS data were analyzed using the Demeter suite of programs^33^. Raw spectra were averaged, calibrated according to the metal foil reference, and normalized in Athena. FEFF fitting was performed using Artemis. FEFF models were constructed from the published crystal structure of 1T-MoS_2_. For the Mo K-edge, no second-shell scattering was observed, so only the first shell was fit with the addition of a single-scattering Mo–O path. Based on previous ICP-MS measurements^28^, it was shown that the Ni@1T-MoS_2_ structure is doped with both Ni and a single O atom associating with each Ni dopant. However, from FEFF fitting, it is not possible to fit first shell Mo–O scattering due to the much larger amplitude of Mo–S scattering in the first shell. Because of this, EXAFS fitting using a Mo–O path with variable CN is not feasible and results in indefinite results. Furthermore, due to the doping percentage of the sample, the CN of Mo–O in the first shell is expected to be <<1 and was therefore not included. There is, however, second-shell Mo–O scattering that was discussed previously^28^. The model utilized is further demonstrated in the calculated FEFF paths (Supplementary Fig. 7). The CN of both the first and second-shell vectors were allowed to vary along with the Debye–Waller factor (σ^2^), Δ*E*_0_, and Δ*R*. For simplicity, the value of *R* is reported rather than Δ*R* which is relative to the input model.

For the Ni K-edge, Mo was substituted for Ni in the 1T-MoS_2_ structure and used directly for fitting. For acidic conditions, a fixed CN of 1 was used for the first shell Ni–O path. For alkaline conditions, Ni–O first shell scattering was accounted for by replacing a S for O in the model and then allowing the CN of each vector to fit. For the catalytic alkaline condition, a Ni atom was added to the model.

## Supplementary information

Supporting Information

Peer Review File

## Data Availability

The datasets generated during and/or analysed during the current study are available from the corresponding author on reasonable request.
