# Supplementary material for: Dynamic evolution and reversibility of single-atom Ni(II) active site in 1T-MoS2 electrocatalysts for hydrogen evolution
Source: Nat Commun. 2020 Aug 17;11:4114. doi: 10.1038/s41467-020-17904-z (PMC7431582; doi:10.1038/s41467-020-17904-z)
Supplement: Supplementary file 1 — Supporting Information [file 41467_2020_17904_MOESM1_ESM.pdf]

Supplementary Information

Dynamic Evolution and Reversibility of Single-atom Ni(II) Active Site in 1T-MoS_2 Electrocatalysts for Hydrogen Evolution

Pattengale et al.

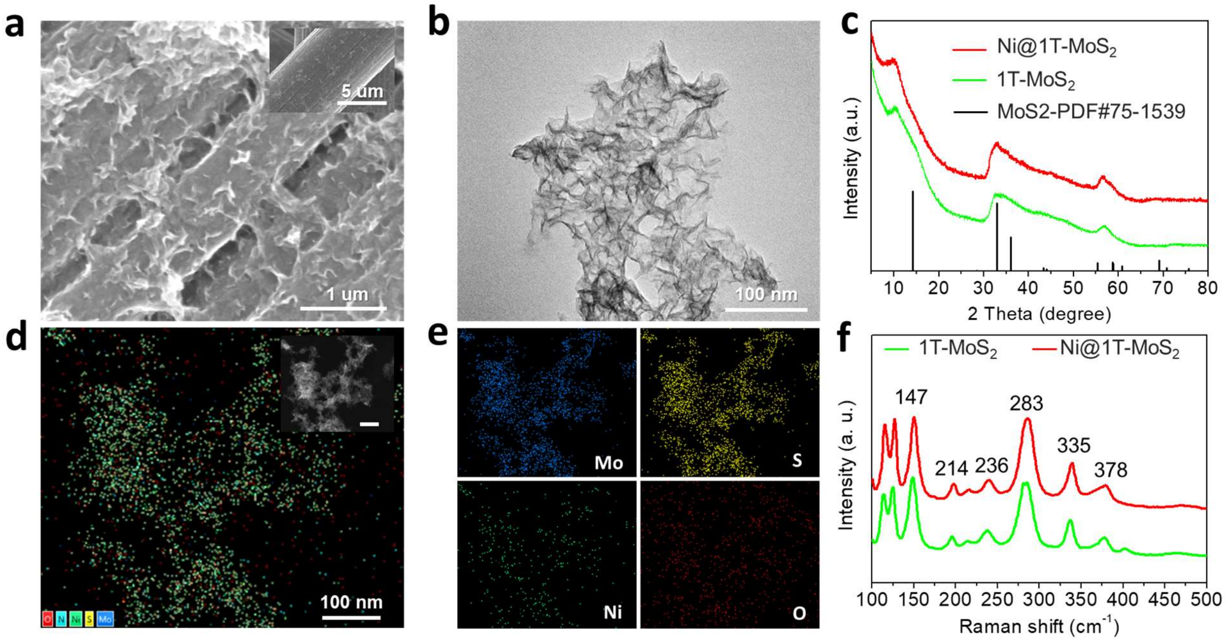

Supplementary Fig. 1 Structure characterizations. (a) SEM image of Ni@1T-MoS_2, scale bar: 200 nm (inset: low magnification SEM image, scale bar: 5 μm). (b) TEM image of Ni@1T-MoS_2 (scale bar: 100 nm). (c) The XRD patterns of the Ni@1T-MoS_2 and 1T-MoS_2 nanosheets. (d) and (e) EDX mappings of Ni@1T-MoS_2 (scale bar: 100 nm). (f) Raman spectra of Ni@1T-MoS_2 and 1T-MoS_2 nanosheets.

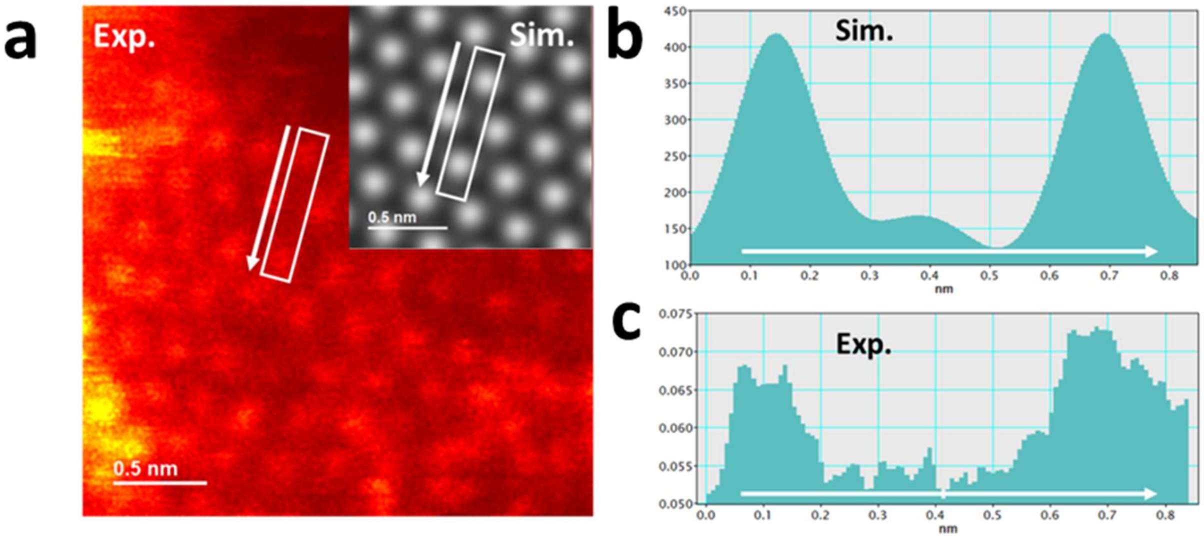

Supplementary Fig. 2 HAADF-STEM characterization of Ni@1T-MoS_2. STEM (a) with the experimental (b) and simulated (c) spectra, which indicates the bright spot is Mo and dark spot is Ni rather than a structural vacant site.

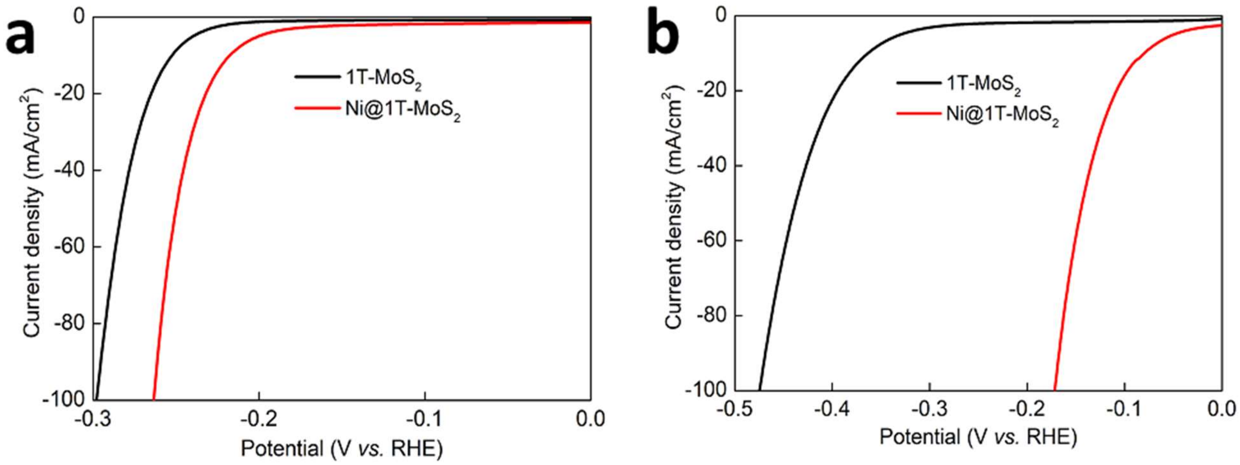

Supplementary Fig. 3 Linear Sweep voltammetry of Ni@1T-MoS_2. Linear sweep voltammetry under acidic 0.5 M H_2SO_4 (a) and alkaline 1M NaOH (b) conditions.

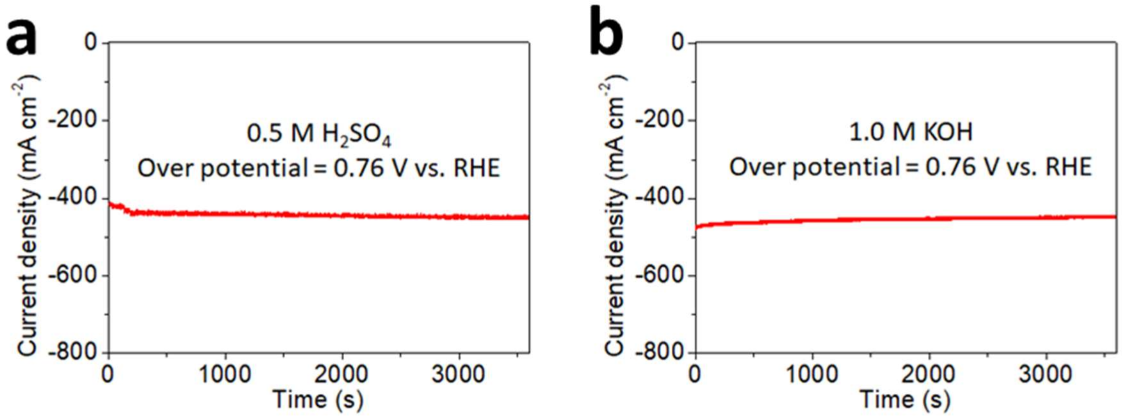

Supplementary Fig. 4 HER stability tests of Ni@1T-MoS$_2$ electrocatalyst. (a) The long-term durability tests in 0.5 M H_2SO_4 at η = 0.76 V for 1 h. (b) the long-term durability tests in 1.0 M KOH at η = 0.76 V for 1 h.

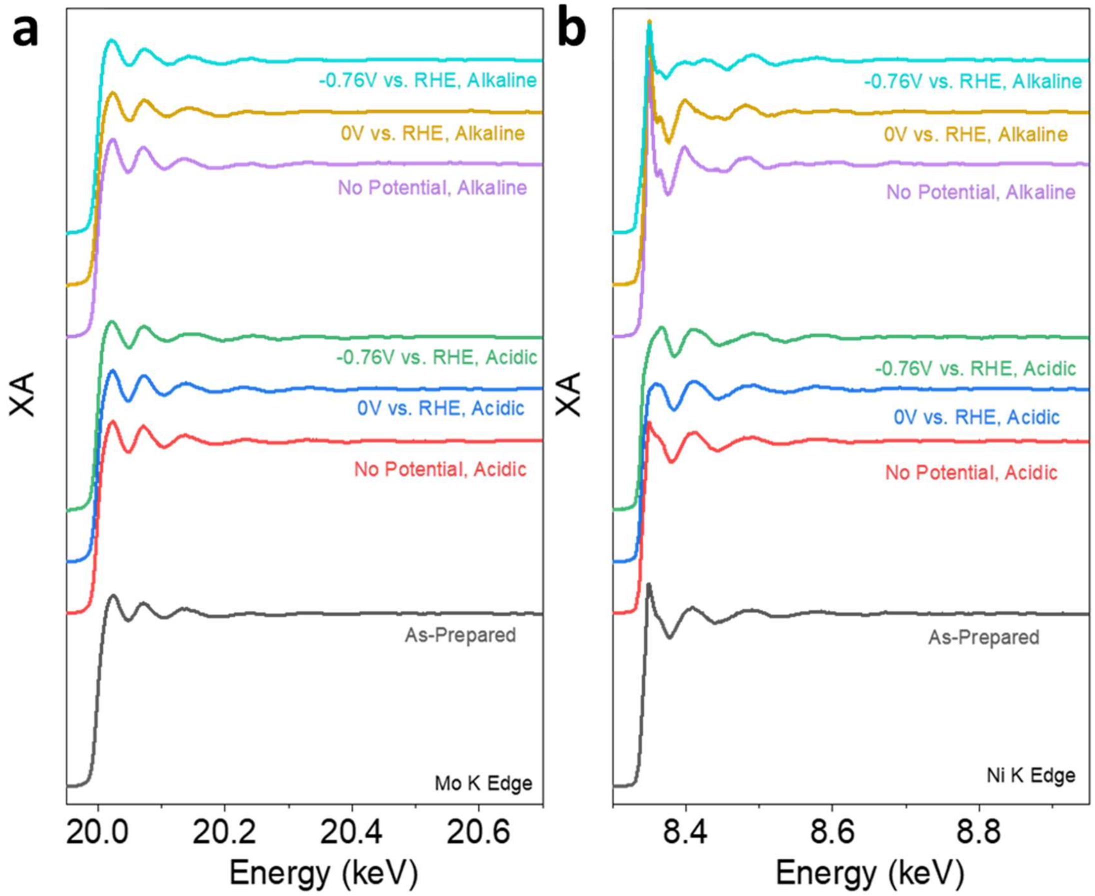

Supplementary Fig. 5 Full EXAFS energy spectra. Spectra in energy space for Mo K-edge (a) and Ni K-edge (b).

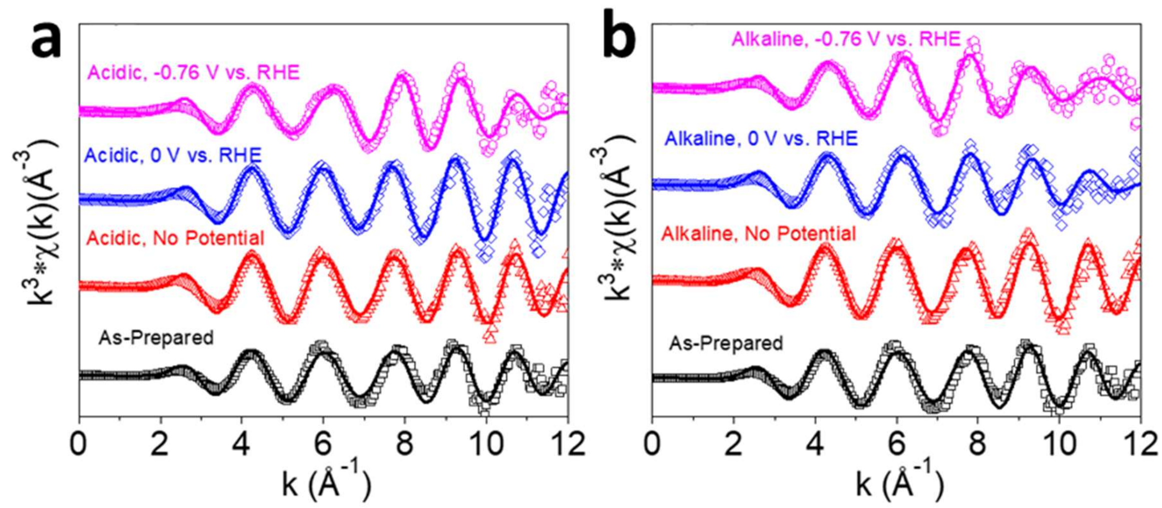

Supplementary Fig. 6 XAS k-space spectra and fits for Mo K-edge. Collected under acidic (a) and alkaline (b) conditions.

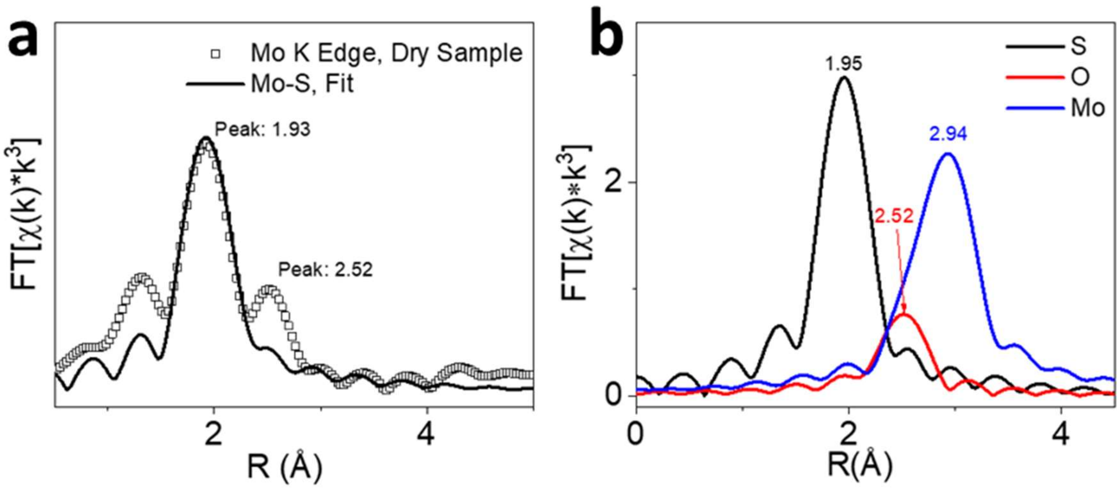

Supplementary Fig. 7 Mo K-edge EXAFS fit model. (a) Shows a fit to the as-prepared dry sample at Mo K-edge using only a Mo-S first shell path. The shoulder at 2.52 Å cannot be fit without an additional path, which was assigned as a Mo-O interaction in the second shell. (b) Shows the calculated individual single scattering FEFF paths from the model from Mo to the atom indicated in the figure legend. The Mo-Mo second shell path is 0.4 Å too long to account for the shoulder feature. The Mo-Mo feature is not typically observed in EXAFS data of 1T-MoS_2, due to Mo being octahedrally coordinated with large S atoms in the first shell.

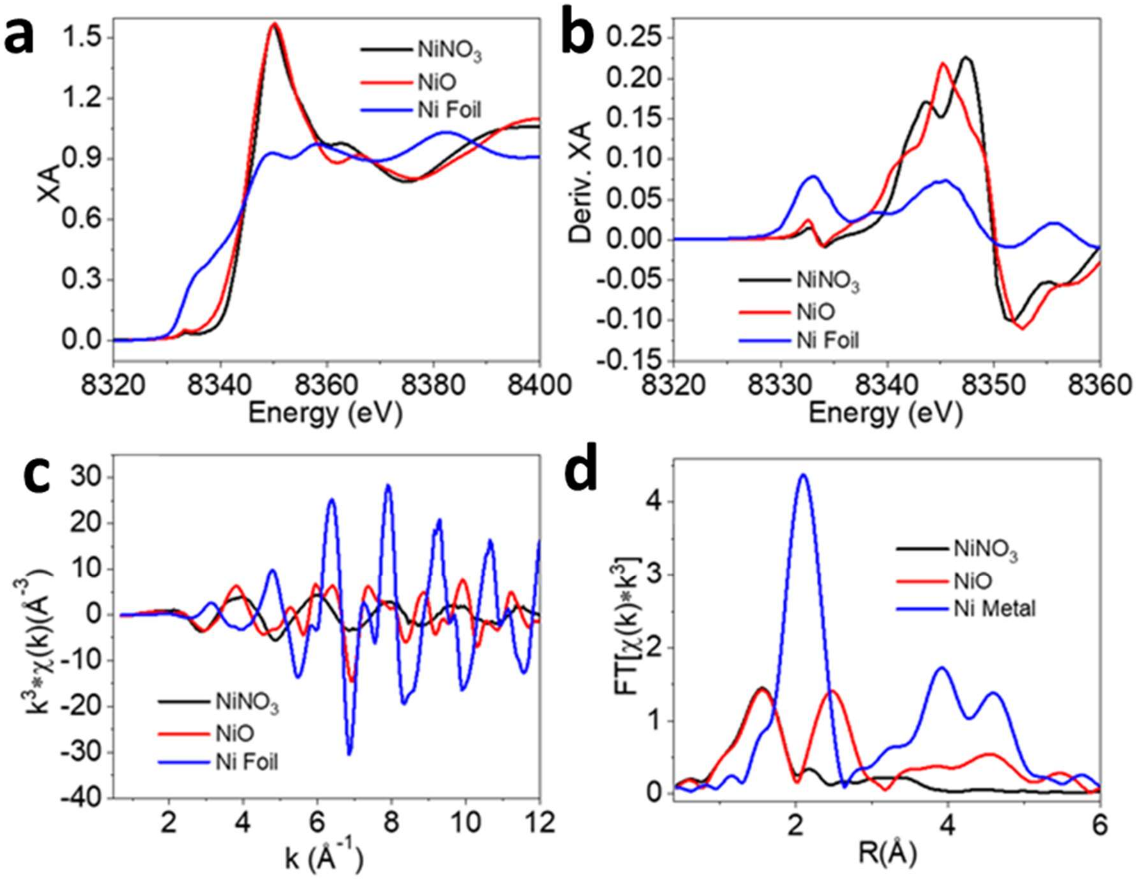

Supplementary Fig. 8 Ni K-edge XAS reference spectra. (a) XANES reference spectra at Ni K edge and (b) first derivative XANES edge. (c) K-space and (d) R-space spectra of references. The first derivative edge positions from (b) are 8333 eV (calibrated) for Ni foil, 8345.19 eV for NiO, and 8347.3 eV for $NiNO_3$. The as-prepared sample has a first derivative peak at 8346.5 eV, which is within the range of edge positions for the Ni^{2+} references.

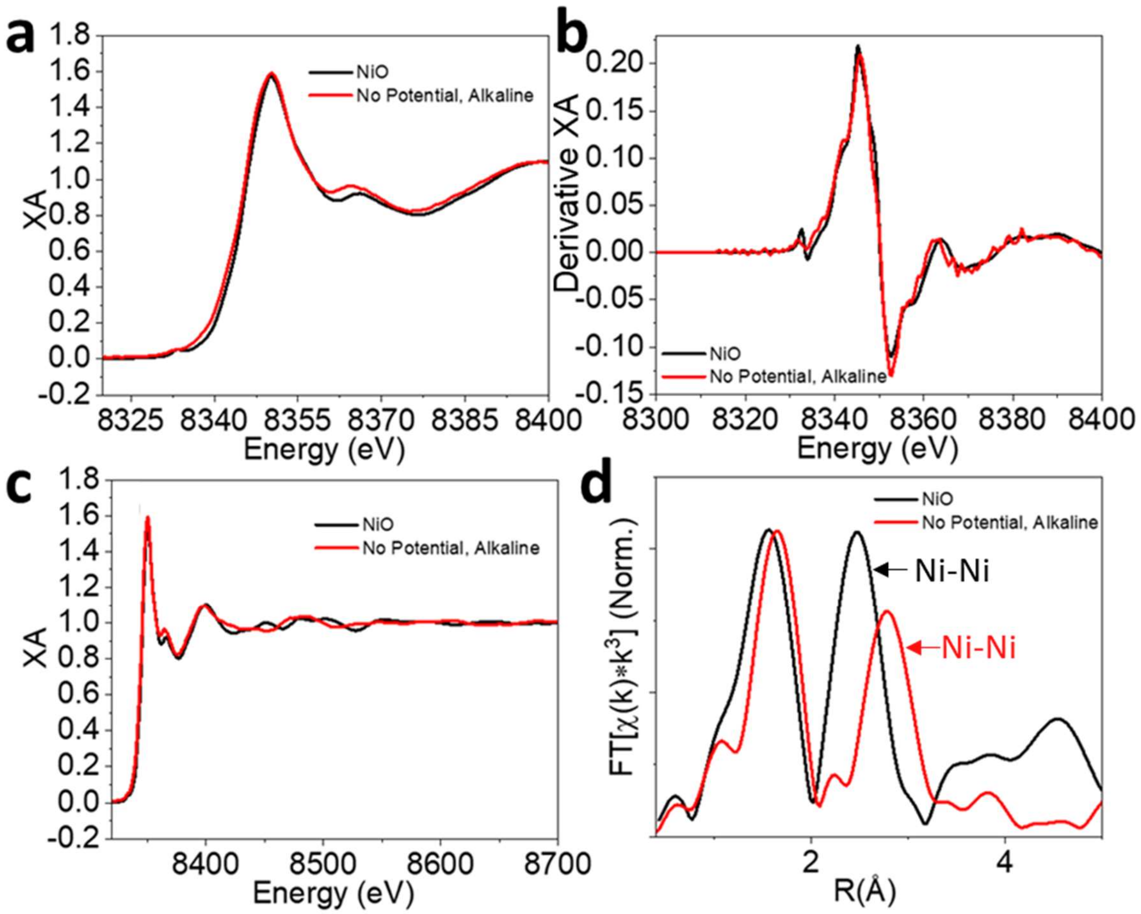

Supplementary Fig. 9 Comparison of NiO reference data to Ni@1T-MoS_2 No Potential, Alkaline data. While the XANES edge (a) and first derivative (b) show similar oxidation state and features, the EXAFS oscillations (c) are significantly different. The R-space spectra were normalized at the first shell scattering feature to yield the comparison in (d) which shows that Ni in Ni@1T-MoS_2 exhibits significantly longer first shell scattering interactions and, furthermore, shows different second shell scattering interactions. It can be interpreted that the Ni-Ni interactions in Ni@1T-MoS_2 are much longer in the second shell, implying that the species formed is not simply NiO, but rather a species still associated with 1T-MoS_2, i.e. NiS_xO_y.

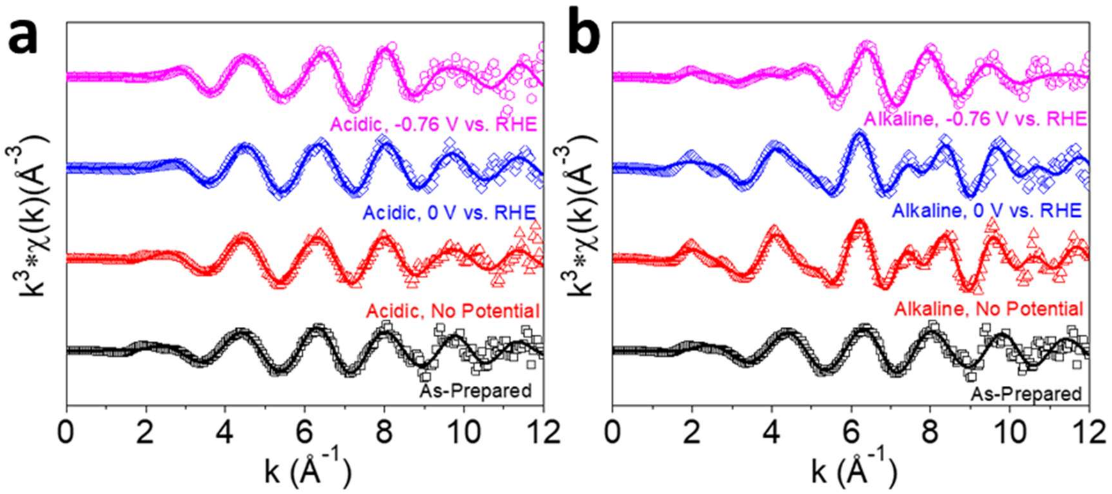

Supplementary Fig. 10 k-space spectra and fits at Ni K-edge. Collected under acidic (a) and alkaline (b) conditions.

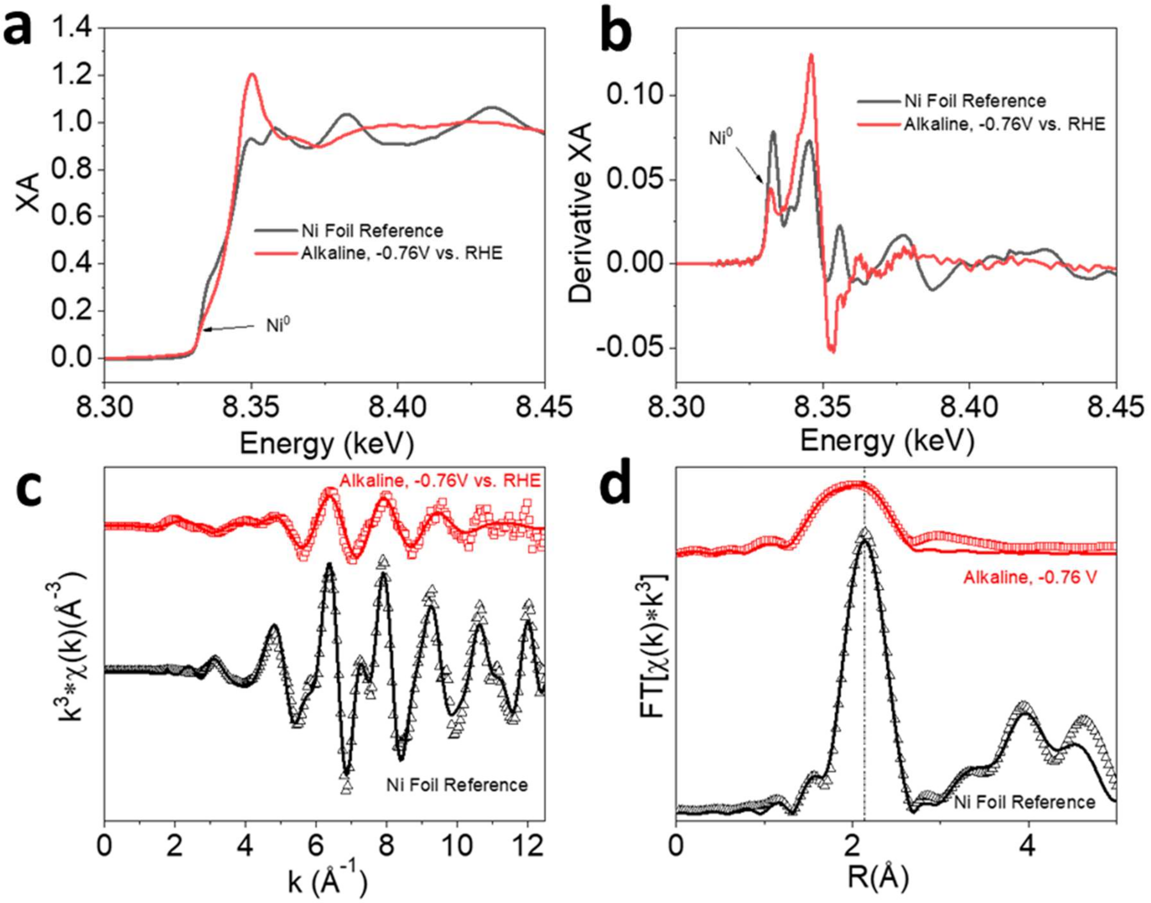

Supplementary Fig. 11 EXAFS comparison to Ni metal reference. Comparison of Ni foil reference XAS spectra to the alkaline, -0.76V vs. RHE condition in XANES region. (a) First derivative of XANES spectra (b), k-space with fits (c) and R-space with fits (d). The corresponding fit parameters for Ni foil reference are shown in Supplementary Table 2.

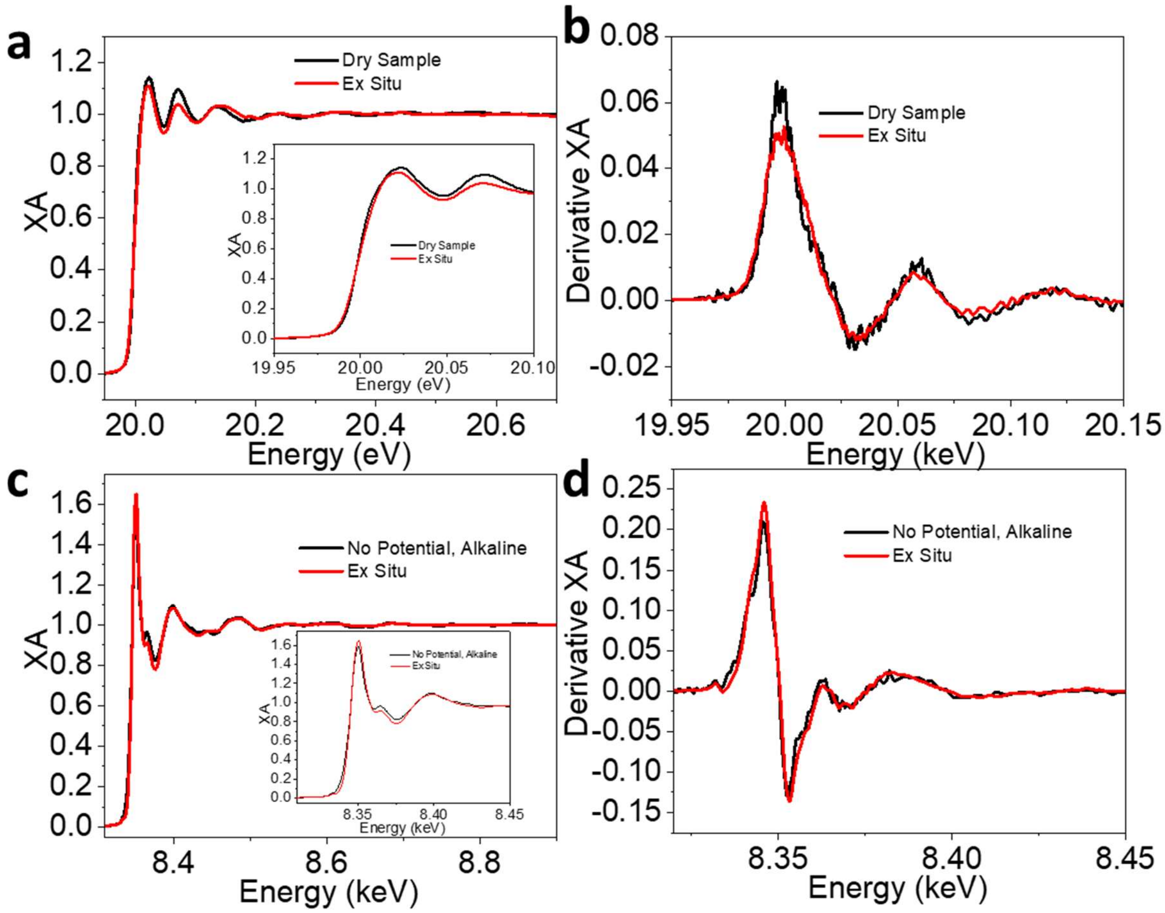

Supplementary Fig. 12 *ex situ* XANES and EXAFS spectra comparisons. Mo K-edge EXAFS (a) and XANES (inset) spectra and first derivative spectra (b) comparing the as-prepared sample to the *ex situ* sample. Ni K-edge EXAFS (c) and XANES (inset) spectra and first derivative spectra (d) comparing the no potential, alkaline sample to the *ex situ* sample. Alkaline conditions were used to generate the *ex situ* sample.

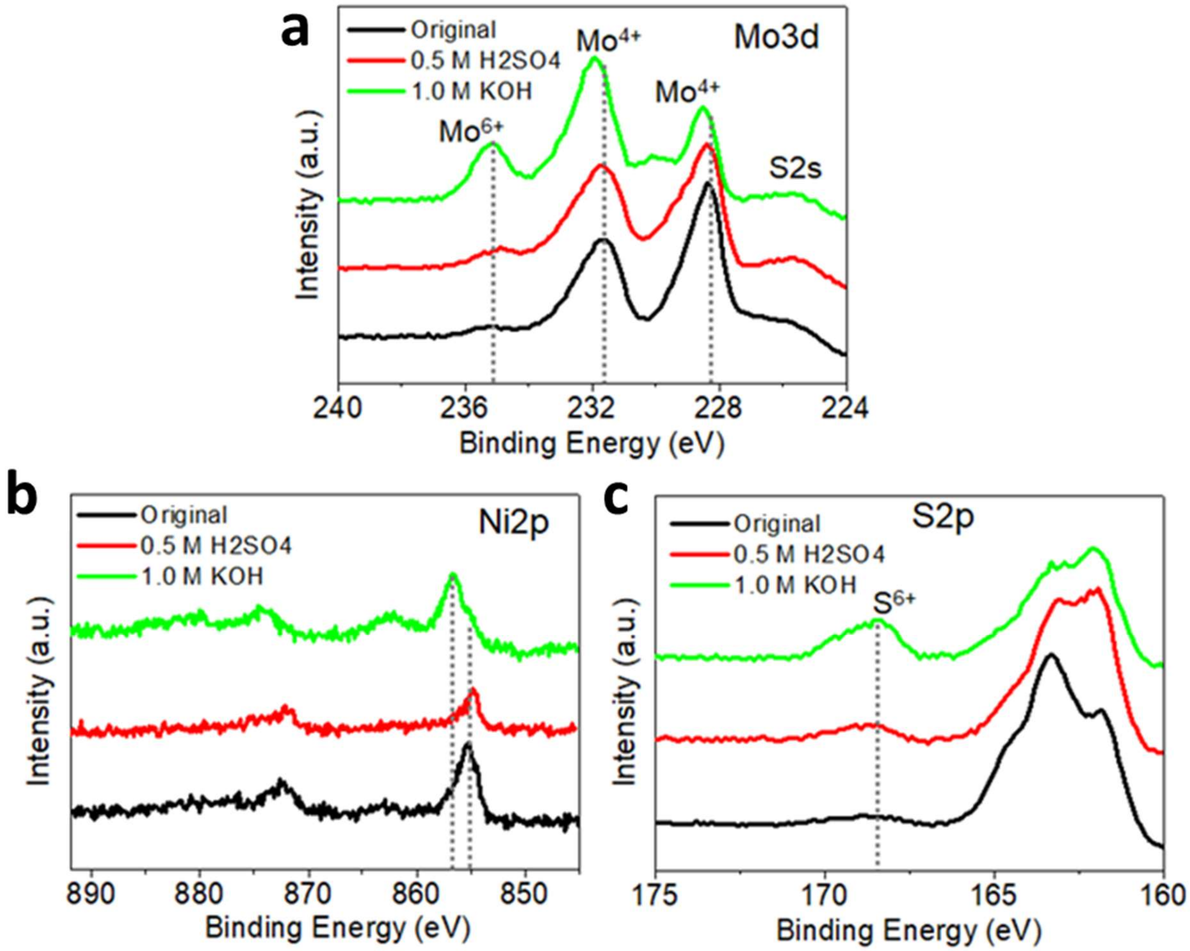

Supplementary Fig. 13 XPS analysis before and after HER stability tests of Ni@1T-MoS$_2$. (a-c) High-resolution XPS signals of (a) Mo3d; (b) Ni2p; (c) S2p for the Ni@1T-MoS$_2$/CFP electrocatalyst.

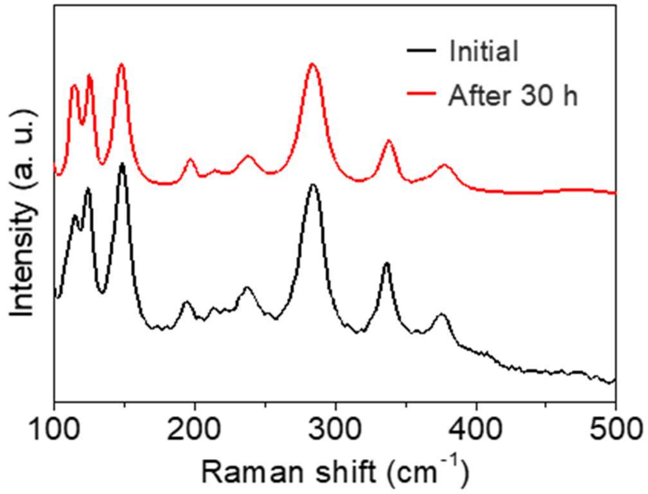

Supplementary Fig. 14 Raman characterization of NiO@1T-MoS_2/CFP. Raman spectra were collected before and after long-term HER stability testing in basic electrolyte. No significant changes are observed and are consistent with the peaks in Supplementary Figure 1.

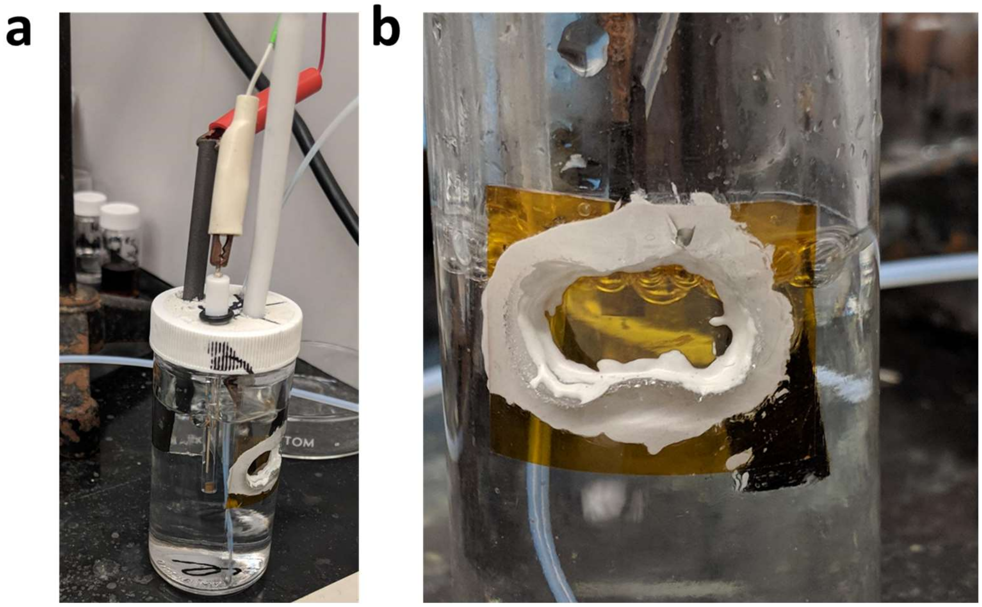

Supplementary Fig. 15 Three-electrode *in situ* electrochemical cell used in this study. (a) The glass vessel has a hole drilled through it. Loctite Hysol epoxy is then used to attach a Kapton window to the inside of the vessel to minimize path length to the electrode. Electrodes (working electrode with clamp for CFP sample, graphite rod counter electrode, and SCE reference electrode (note: Ag/AgCl shown in example picture)) are inserted through the cap and through a Viton sheet gasket. A Teflon tube is inserted into the bottom of the cell for purging. A close up (b) shows the Kapton window area where the sample is positioned during measurement.

Supplementary Table 1 FEFF fitting parameters for Mo-K edge *in situ* XAS data.

Condition	Vector[a]	CN[b]	σ^2 (Å)^{2} [c]	ΔE_0 (eV)	R (Å)[d]
As-Prepared	Mo-S	4.17	0.006	-1.95	2.44
	Mo-O	2.81	0.001	-9.51	2.88
No Potential, Acidic	Mo-S	4.98	0.005	-1.48	2.44
	Mo-O	3.45	0.001	-8.08	2.87
0V vs. RHE, Acidic	Mo-S	4.55	0.004	0.38	2.46
	Mo-O	3.47	0.001	-6.78	2.89
-0.76V vs. RHE, Acidic	Mo-S	4.06	0.006	-2.77	2.40
	Mo-O	3.80	0.001	-5.25	2.91
No Potential, Alkaline	Mo-S	4.81	0.005	-6.82	2.45
	Mo-O	3.19	0.001	-10.1	2.86
0V vs. RHE, Alkaline	Mo-S	4.78	0.008	-2.30	2.42
	Mo-O	1.88	0.001	-5.79	2.92
-0.76V vs. RHE, Alkaline	Mo-S	4.17	0.007	-3.21	2.41
	Mo-O	1.58	0.001	-2.00	2.99

[a] Mo-S is a first shell parameter while Mo-O is a second shell parameter. [b] Coordination Number, Uncertainty ± 0.5. [c] Uncertainty ± 0.001 Å^2. [d] Uncertainty ± 0.02 Å.

Supplementary Table 2 Reference Ni Foil FEFF fitting parameters.

Vector	Coordination Number	σ^2 (Å)^2	ΔE_0 (eV)	R (Å)
Ni-Ni (1st shell)	12	0.008	-6.13	2.49
Ni-Ni (2nd shell)	6	0.010	4.28	3.59
Ni-Ni (3rd shell)	24	0.012	-5.16	4.33
Ni-Ni-Ni	96	0.001	11.46	4.67

Supplementary Table 3 ICP-MS results of the electrolytes before and after HER stability tests of Ni@1TMoS$_2$ electrocatalyst.

Elements contents (μg/mL)	Before HER stability tests in 0.5 M H_2SO_4	After HER stability tests in 0.5 M H_2SO_4	Before HER stability tests in 1.0 M KOH	After HER stability tests in 1.0 M KOH
Mo	<0.1	<0.1	<0.1	0.4713
Ni	<0.1	<0.1	<0.1	<0.1

Supplementary Table 4 ***Ex Situ*** **XAS FEFF fitting parameters.**

Edge	Vector	CN[b]	σ^2 (Å)^{2} [c]	ΔE_0 (eV)	R (Å)[d]
	Ni-S	1.82	0.015	0.660	2.23
Ni K	Ni-O	4.30	0.006	0.660	2.05
	Ni-Ni	4.40	0.010	6.048	3.09
Mo K	Mo-S	5.20	0.009	-5.67	2.38
	Mo-O	2.24	0.001	-3.88	2.87
