# Supplementary material for: Dynamic evolution and reversibility of single-atom Ni(II) active site in 1T-MoS2 electrocatalysts for hydrogen evolution
Source: Nat Commun. 2020 Aug 17;11:4114. doi: 10.1038/s41467-020-17904-z (PMC7431582; doi:10.1038/s41467-020-17904-z)
Supplement: Supplementary file 2 — Peer Review File [file 41467_2020_17904_MOESM2_ESM.docx]

Peer Review Information

**Journal:** Nature Communications

**Manuscript title: Dynamic Evolution and Reversibility of Single-atom Ni(II) Active Site in 1T-MoS_2_ Electrocatalysts for Hydrogen Evolution**

**Corresponding author name(s):** Prof Jier Huang

**Editorial notes:**

**none**

**Reviewer comments & decisions:**

**Reviewer comments, first version:**

Reviewer #1 (Remarks to the Author):

I recommend major revision.
In the above paper the authors have prepared Ni doped MoS2 on conductive carbon fibre paper and have tried to establish realisation of single atom Ni catalyst in basal plane of MoS2 by several complementary technique. The focus of the present paper is however to characterise the samples by XANES and EXAFS measurements at Ni and Mo K edges in both ex-situ and in-situ conditions. The measurements have been carried out both on as-prepared catalyst and catalysts during electrochemical reactions under alkaline and acidic conditions and under application of bais. The main finding of this paper is that under alkaline condition NiO gets reduced to metallic Ni form and work as active catalyst site at the basal plane of MoS2 and it reverts back to oxide phase after the catalytic reaction.

The paper has significant importance in understanding the structure-activity correlation of single atom Ni catalyst in Hydrogen evolution reactions and thus has significant importance on a technology front of current interest and hence can be considered for publication.

I have however some serious objections on the presentation and interpretation of the EXAFS fitting results given in the paper which are summarised below:

(i) Though uncertainty in bond length (R) and Debye-Waller factor (σ^2^) has been given in Table-1 and Table-2, no uncertainty value is mentioned in the best fit result of co-ordination number (CN). I know this is dicey issue for EXAFS professionals, however the authors could have given the uncertainty values obtained in FEFF fitting output itself to get a feeling since many of the conclusions of the paper depends on this.
(ii) The first peak of FT [(k)k^3^] vs. R plots of Mo K-edge data has been fitted assuming Mo-O and Mo-S shells. However, the authors must show their theoretical positions in the FT [(k)k^3^] vs. R plot and establish undoubtedly that both the shells are required to obtain the best fit.
(iii) There is abrupt change in the Mo-S and Mo-O coordination number as catalysts is immersed in alkaline and acidic solution and as well as by applying bias. No explanation has been provided for these changes.
(iv) Table-1 also shows that Mo-S bond length reduces significantly on application of -0.76 V vs. RHE both in alkaline and acidic media (which the authors have mentioned also in line no.s 144-146), however, no explanation of that is also given.
(v) During fitting of Ni K edge FT [(k)k^3^] vs. R data also Ni-Mo path has been taken, however in the plots of as-prepared and acidic samples hardly any peak is visible at around 3 Ả. Here also the authors have to establish this with more proof. If it is there why it has not been taken in case of the alkaline sample? It is getting submerged in the large peak of Ni-Ni? More explanation is needed on this.
(vi) The significant change in the FT [(k)k^3^] vs. R plot when a catalytic potential of -0.76 V is applied in alkaline medium, the peak becomes very broad and fitting shows that the Ni-Ni bond length which is otherwise at 3.1Ả becomes 2.53Ả to fit this broad peak. It needs clear explanation. How Ni-Ni peak can get shortened so much?
(vii) I am also very much perplexed about fig. 5 where the authors have claimed that Ni has gone back to Ni-O phase from metallic Ni phase after catalytic reaction. But then why the strong peak around 2.75Ả is appear in the FT [(k)k^3^] vs. R plot of the ex-situ sample which if I have understood correctly is due to Ni-Ni shell.

(viii) Finally I am not sure why the authors claim that cluster of 10 Ni atoms or less is formed in line no. 260?

I feel the present paper cannot be accepted unless the above issues are addressed. I would like to review the revised version.

Reviewer #2 (Remarks to the Author):

This manuscript reports in situ XAS characterizations of Ni single atoms doped on basal plane of 1T-MoS2, and the HER catalytic mechanisms in both acidic and alkaline solutions, especially the role of Ni single atoms were thoroughly investigated. It was found that under acidic conditions the Ni atoms remains stable as its native structure (status) while in alkaline media, a reversible feature of metallic Ni species (Ni0) is observed. The Ni-doped 1T-MoS2 catalyst system has been reported and other reports on similar catalysts also exist, but the value of the current work is the in situ studies to understand the true roles of single atom as the active sites in MoS2 toward HER. Therefore, I recommend it to be published in Nature Comm. However, there are some major issues should be addressed:
1. Do you have clear evidence that Mo does not go to Mo (0), as Ni does in the alkaline HER conditions? It sounds like the Mo foil data were collected as reference but you did not specifically compare the EXAFS data. I am asking this because in reality, NiMo alloy has been the benchmark HER catalyst in alkaline conditions.
2. The authors have not considered the defects and vacancies situations in their solution synthesized/converted 1T-MoS2 (which are prone to have defects). These previous reports (J. Am. Chem. Soc. 2016, 138, 7965, J. Am. Chem. Soc. 2016, 138, 16632, the cited ref. 5) showed that vacancy defects are very important to catalytic activity. Could it be possible that some current data already reveal something about the defects that were not paid attention to? If vacancies were very prevalent around the Mo metal, I think we would see a <6 coordination number from the fitting. The authors do see a <6 CN in the first-shell fitting for Mo, but they say there is an additional Mo-O path that is convoluted and hard to fit.
3. The authors do not seem to adequately explain why the Ni is dark and the Mo are bright, when they explicitly point out that literature demonstrates the opposite. It seems like electrons should more easily pass through the low Z element. The zone axis is not stated. It seems the cited papers also support that this trend should be opposite. Could these be vacancy sites instead? A zoom out TEM image should also be shown to demonstrate the sample region. Does the density of nickel active sites make a difference, and should this be further characterized? No attempt at characterizing the density of nickel active sites of amount of nickel in the structure appears to be made. Figure S10 and the implications of when Ni atoms become less isolated should be mentioned in the text along with Figure 2.
4. Since the oxygen element is involved in precursor, the influence (contribution) from oxygen for the properties can’t be completely excluded and in fact, the authors have claimed that although no Mo-O scattering in first shell was observed, ICP analysis with a higher resolution demonstrated the oxygen doping in MoS2. Please provide more discussion on this.
5. Are the XRD and Raman results shown in Figure S2c and f shown for the same NiO@1T-MoS2 catalyst grown directly on CFP or is this a powder sample? There’s no apparent peak in the XRD as a result of the CFP, and the crystallographic labels for MoS2 are not included, and the corresponding characterization of the 1T-MoS2 catalyst is also not included.
6. The indicated “shift” in the first shell in acidic media in Figure 3c seems fairly minute – seemingly only barely outside of the error of the fitting according to Table 1? Perhaps the values from the fits could be somehow included in the figure to more effectively communicate the changes, as they are difficult to observe from the data.
7. The discussion of 1T to 2H transformation could be more convincing with before and after ex-situ XRD or SAED patterns.
8. The conclusions derived from Figure 4 and 5 make sense, but lead to the questions about the absence of a major Ni-Mo scattering path in the pristine sample. The authors state that an equivocal Mo-Ni scattering path doesn’t occur under applied potential, but given that the Ni atoms are sparsely dispersed across the sample, this being an intense enough scattering pathway to observe for Mo seems unlikely anyways. If Mo-Mo and Ni-Mo is always too weak to be observed, which seems like the case, perhaps Ni-Mo shouldn’t be attempted to fit in the acidic Ni K-edge results.
9. Both of the cited examples of metal lability in graphene are under electron irradiation. A more relevant example of surface reconstruction could be more helpful?
10. In Figure S4, the long-term stability for Ni@1T-MoS2 seems not very good. If this indicated the instability of Ni atoms in MoS2 matrix during the HER test in both electrolytes? If the Ni single atoms will migrate during the HER test? If it is possible, I think there may be some difference in in situ XAS spectra measurement as a function of the test time.
11. The author claimed the reversible feature of Ni single atom in alkaline conditions can be observed from immersion in electrolyte and the application of a catalytic potential. If this behavior could be well repeated after a long-term test.
12. Other examples of TM-atom doped MoS2 catalysts: ACS Energy Lett., 2018, 3, 2685. DOI: 10.1021/acsenergylett.8b01567. This one specifically compares the acidic vs. alkaline HER on MoSe2 and MoS2: ACS Energy Lett., 2017, 2 , 2234.
13. Ref. 28 and 30 are duplicates. In general, there could be more careful reference citation practice in general.


Reviewer #3 (Remarks to the Author):

The paper by Patengale et al. describes in situ and operando X-ray absorption studies, which aim is to demonstrate the contribution a Ni single atome electrocatalyst on T-MoS2 for hydrogen evolution. Although the topic is of interest and the use of in situ XAS is appropriate, the conclusions are not supported by the data collected. More specifically, the collected data are not analysed in sufficient depth to allow drawing such conclusions. As a consequence, I have to reject this paper from Nature communications. Besides, I am not convinced that a communication is the best format for such a paper, which would benefit from a more thorough analysis and discussion of the data collected.

Here are several points that justify my decision and could help authors improve their paper:

- In the abstract and introduction, the term “native environment” is used twice to describe the structure of the nickel ion understander catalytic conditions in acidic conditions. This is a bad start, since this vague term just suggests that the authors cannot describe precisely what this environment exactly is.

- The synthesis description mentions the synthesis of POMs with Fe, Co or Ni as hetero-anion, but only the Ni-substituted one is studied in the paper. Why mention the other metals? Also, it would be very important to synthesise the same POM without any (deliberate) addition of nickel, in order to assess the actual role of nickel in catalyst and in the spectroscopy.

- Line 74-75, we understand that the material has been deposited on a carbon paper, but there is absolutely no experimental details on this procedure. It need to be provided, at least in the SI.

- line 77: the word “inevitably” is incorrectly used, as often throughout the aper and suggests that the authors use characterisation techniques to demonstrate something that they have already concluded about.

- line 86-87: it is assumed that the lower intensity observed on one spot of the STEM image is a clear proof of Nickel insertion, but there might be other explanations for this lower intensity, such as a simple defect in the MoS2 network. The authors should comment about this possibility.

- the paragraph between lines 88 and 95 mentions the electrochemical performances of the Ni@T-MoS2 material with respect to T-MoS2. How was the reference T-MoS2 prepared? Was it prepared and studied by the authors or are the data collected from another work? This is not clear in the curent version of the paper.

- I am a bit skeptical about the data collected under in situ conditions. The setup described by the authors with a kapton membrane facing the working electrode is too rudimental to be completely confident in the data collected. The amount of nickel in the sample is supposed to be very low, so any contamination from adventitious nickel (in the glue, in the plastic or anywhere else) could compromise the quality and hence the significance of the data. The authors should at least provide blank XAS spectra of a setup where everything is equal, except for the nickel substitution in the 1T-MoS2. The carbon paper itself can often contain metallic traces. Ideally, another setup with the working electrode facing the incident beam should be used. There are plenty of examples of such setup, such as described in King et al. J. Phys. Chem. C 2019, 123, 28533−28549. There are plenty of other papers using electrochemical cells for in situ X-ray absorption that are much more evolved (yet not that complicated) than the one used here.

- On figure 3, the XANES is not mandatory, since there is nothing to learn from it. It could be sent to the SI. On figure 3c, there are plain lines and symbols, what do they correspond to? Fits and data? These kind of issues should not be the work of a reviewer but of co-authors! Please proof-read your papers before sending papers for publication!

- Line 148: I understand from this sentence that the shortening of the Mo-S bond is due to the fact that nickel is the active in Ni@1T-MoS2. How does that relate? Why would the shortening of an Mo-S bond under catalytic conditions make more sense in a Ni@1T-MoS2 than in amorphous MoSx? This assumption should be explained with sound arguments.

- The analysis of the XANES data presented on figure 4a is a very weak point of the paper. The authors basically claim that the spectra of the as prepared, no potential in acid, 0V and catalytic conditions are identical. Or at least that the changes are so minimal that they don’t mean anything. This is either a poor knowledge of X-ray absorption spectroscopy or a deliberate intention to hide what is not understood in order to orient the results of their study. In both cases, it is a serious problem! The assumption that the species does not change is based on the first derivative of the XANES spectra, which indeed do not show a shift of the main edge position. But XANES is not just about edge position, it can be clearly seen that the spectra are not identical, both from the original spectra and from the derivatives. These differences need to be discussed, even if their origins cannot be clearly identified.

- One main problem of this study associated with these data is the lack of reference samples. Any good XAS study is based on the comparison with model compounds (unless high level calculations are provided). Why didn’t the author measure and report the XANES and EXAFS spectra of a nickel foil (it is shown in the SI), of NiO, Ni(OH)2, NiS, Ni3S2, NiSO4, the original NiMo POM, etc…This is the basic of any XANES study and I am actually shocked that the authors sent a paper for publication without that, at least in the SI. If reference spectra had been measured, then linear combination fittings could have been performed and a first approximation of the composition of the species could have been obtained. This is true for both the acidic and basic conditions.

- The EXAFS fits should be performed in a more comparative way. A fit is not good or bad on its own, it should be compared to another fit, with different parameters. This is lacking in this study. For example, the fit of the species under alkaline conditions is performed with both Ni-O and Ni-S scatterrers, it should be compared to fits where only one of the scatterrers is used, so as to see which one is best.

- The uncertainties in table 2 should be given. Not as a general value in the caption as is done in note (b), but in brackets for each compute value. This is an important aspect of fits which also gives a sense of how good the fit is.

- The migration of nickel species to form metallic nickel during the catalytic experiment is proposed on the basis of EXAFS and TEM data. Figure S10 shows a TEM image where two supposed nickel sites are next to each other, suggesting the possible formation of nickel clusters. But this image was taken from the as prepared material, which is not relevant at all, wince there is no or little metallic character in the as prepared material. Also two adjacent nickel atoms should give an average coordination number of 1 for the Ni-Ni shell observed at R’=2.0 Å, which does not seem to be the case given the intensity of the peak at the position (table 2 indicates a value of 2).

- The reversibility of the metallic nickel formation would be a very interesting thing if t was clearly demonstrated. But why were data only taken ex situ? It would be much more sensible to collect data at 0V after catalysis, or at open circuit.

- line 242: in the caption of figure 5, what does “the applicable results “mean?

- there is not, anywhere in the paper, any evidence for an interaction between the nickel ions and the 1T-MoS2 sheets. It is very well possible that all nickel ions detach from their substrate, migrate, and forma nanoparticles. These nanoparticles would in turn oxidise under alkaline conditions to NiO. This is actually strongly suggested by the fits under alkaline conditions (no potential or alkaline), which show N numbers >4 (corresponding to a cluster of at least 13 atoms). This value does not corresponds to the proposed structure in figure 6 (even if it is only schematic), where the N value should be ca. 1.3. These particles could then be partially reduced to metallic nickel under cathodic potential, and reoxidised to nickel oxide when brought to air. I don’t see any experimental evidence in the current version of the paper to rule out this hypothesis.

- The conclusion of the article is far from a sound scientific reasoning. After assuming the reversibility of their active species from nickel oxide to metallic nickel and back, the authors surmise that the nickel species can, on its own, be active at the interface of Ni@1T-MoS2. This is a very long stretch! The reversibility of the process (if proven) does not imply that the nickel at the interface is active, it is rather the opposite! It needs to change its structure (in the authors opinion migrate to form small clusters, in my opinion desorb to form nanoparticles) to be active.

- Information about the synthesis and chemicals in the experimental part are too succinct. There is a long sentence from line 297 to 300, which does not make any sense to me. The method to deposit the catalysts on the carbon electrode is not properly described, which is very important. Also, the type of carbon electrode (supplier) is not indicated, which can be critical since these materials sometimes have metallic impurities. The origins of the general chemicals used are not given, which is again important because they might contain metallic impurities. (it is well know since the famous paper from Boettcher, JACS 2012, that most chemicals contain Fe impurities that can participate to catalytic activity).

All in all, this paper presents two main serious issues:
1. The lack of care in conducting the experiments presented (missing reference spectra, experimental conditions, reference molybdenum samples analysed) and in analysing them (linear fit combinations, comparative fits). Several points have been raised by the reviewer, which should have been noticed and discussed by co-authors.
2. There seems to be a serious mismatch between the experimental data collected and the conclusions drawn from them. The authors desperately want to show that nickel SAC are activating the base plane of MoS2, while many other hypothesis arise from the data that they present.

**Author rebuttal, first version:**

***Reviewer 1***

*In the above paper the authors have prepared Ni doped MoS2 on conductive carbon fibre paper and have tried to establish realisation of single atom Ni catalyst in basal plane of MoS2 by several complementary technique. The focus of the present paper is however to characterize the samples by XANES and EXAFS measurements at Ni and Mo K edges in both ex-situ and in-situ conditions. The measurements have been carried out both on as-prepared catalyst and catalysts during electrochemical reactions under alkaline and acidic conditions and under application of bais. The main finding of this paper is that under alkaline condition NiO gets reduced to metallic Ni form and work as active catalyst site at the basal plane of MoS2 and it reverts back to oxide phase after the catalytic reaction. The paper has significant importance in understanding the structure-activity correlation of single atom Ni catalyst in Hydrogen evolution reactions and thus has significant importance on a technology front of current interest and hence can be considered for publication. I have however some serious objections on the presentation and interpretation of the EXAFS fitting results given in the paper which are summarised below:*

*(i) Though uncertainty in bond length (R) and Debye-Waller factor (σ^2^) has been given in Table-1 and Table-2, no uncertainty value is mentioned in the best fit result of coordination number (CN). I know this is dicey issue for EXAFS professionals, however the authors could have given the uncertainty values obtained in FEFF fitting output itself to get a feeling since many of the conclusions of the paper depends on this.*

**Author Response:** We thank the reviewer for raising this point. As the reviewer mentions, obtaining reliable (i.e. small uncertainty range) coordination numbers is quite difficult, while still allowing as many other parameters as possible to fit. The coordination number is highly correlated with the Debye Waller factor that accounts for static and dynamic disorder. We find it most appropriate to report a single uncertainty value that corresponds to the typical fit uncertainty using the models employed. For the Mo K-edge data, which all utilizes the same model and fits very similar data, we find a typical fitting uncertainty of ± 0.5 for the first shell with the Debye Waller factor allowed to fit, and again ± 0.5 for the second shell with the Debye Waller factor fixed at its reasonable minimum value of 0.001. For the Ni K edge data, we find similar uncertainty (± 0.5). This uncertainty value is conservative and reflects that coordination numbers must be significantly different to draw any definite conclusions. The uncertainty value of ±0.5 has now been added into the notation below Table 1 and also Table 2 in the revised manuscript. Note that our discussions and conclusions are focused mainly on the large changes observed and therefore our revisions to include this uncertainty value do not change the conclusions of the work.

*(ii) The first peak of FT [χ(k)k3] vs. R plots of Mo K-edge data has been fitted assuming Mo-O and Mo-S shells. However, the authors must show their theoretical positions in the FT [χ(k)k3] vs. R plot and establish undoubtedly that both the shells are required to obtain the best fit.*

**Author Response:** We thank the reviewer for the comment. We first note that for the Mo K-edge, the Mo-S path is a first shell parameter while the shoulder on the first shell feature is assigned as a Mo-O path. This assignment was made in our previous work on both undoped 1T-MoS_2_ and Ni@1T-MoS_2_, including other transition metal doped analogues [Huang, Y. et al. *Nature Communications*, **2019**, *10*, 982, Ref. 28] and was, again, required to fit the data in this work. Without the Mo-O path, the data cannot be properly modeled. It is not assigned to a second shell Mo-Mo interaction because the predicted Mo-Mo peak based on the crystal structure is at 2.94 Å whereas the shoulder observed in the data peaks at 2.51 Å, nearly half of an Angstrom too short. Attempting to fit this shoulder with a Mo-Mo path further results in an unrealistically short Mo-Mo distance for the structure. The calculated paths from the model are shown compared in the top figure below.

We also note that, in addition to the characterizations in the manuscript, we are certain of the phase of MoS_2_ because the XAS data for 2H-MoS_2_ is extremely different. The bottom set of figures and description is excerpted from the Supporting Information of our previous work [Huang, Y. et al. *Nature Communications*, **2019**, *10*, 982, Ref. 28]. Note that the different local structure of Mo in the 2H phase allows clear observation of second shell Mo-Mo interactions. Based on the previous work and these further figures, we hope that our choice of fitting model is clearer. We have added these figures to the SI (Figure S7) to better inform the reader as to the rationale for the fit model utilized. We again thank the reviewer for suggesting this improvement.

**Figure S7.** (a) Shows a fit to the as-prepared dry sample at Mo K-edge using only a Mo-S first shell path. The shoulder at 2.52 Å cannot be fit without an additional path, which was assigned as a Mo-O interaction in the second shell. (b) Shows the calculated individual single scattering FEFF paths from the model from Mo to the atom indicated in the figure legend. The Mo-Mo second shell path is 0.4 Å too long to account for the shoulder feature. The Mo-Mo feature is not typically observed in EXAFS data of 1T-MoS_2_, due to Mo being octahedrally coordinated with large S atoms in the first shell.

*(iii) There is abrupt change in the Mo-S and Mo-O coordination number as catalysts is immersed in alkaline and acidic solution and as well as by applying bias. No explanation has been provided for these changes.*

**Author Response:** We thank the reviewer for the insightful comments. In the original manuscript we provided the following statement:

*“there is an observable difference in the intensity of the first shell peak which is corroborated by the fitting results. The two parameters mainly affecting the intensity are the coordination number and the Debye-Waller factor (σ^2^), which is related to static and dynamic disorder in the lattice. Because σ^2^ is not significantly different between the as-prepared and immersed samples, the intensity difference can be interpreted as a change in the average coordination number of Mo atoms. The changes observed are therefore likely the coordination of solvent to Mo.”*

Given the uncertainties in coordination number, we are unsure exactly what structure changes are observed at Mo, however we suggest that there are some surface states that were undercoordinated in the dry as-prepared sample that became coordinated with aquo or hydroxyl ligands in solution. We have added this possible explanation to the revised manuscript (line 164-167, Page 10). We have kept the explanation in the manuscript broad to not suggest specific species that we cannot comment on further.

*(iv) Table-1 also shows that Mo-S bond length reduces significantly on application of -0.76 V*

*vs. RHE both in alkaline and acidic media (which the authors have mentioned also in line*

*no’s 144-146), however, no explanation of that is also given.*

**Author Response:** We agree that, at this point in the manuscript, there is not clear reason for this contraction of the local structure of Mo. No significant changes in the oxidation state or electronic structure of Mo are observed in XANES. This implies that some change in the structure is due to another factor. This contraction does, however, agree with structure changes observed at the Ni K-edge further in the manuscript, as Ni-Mo second shell scattering becomes shorter under acidic conditions. Under alkaline conditions, very significant changes in the Ni local structure are observed which likely impact the Mo results.

There exists a layer of difficulty in directly comparing the Ni K-edge results to the Mo K-edge results. Due to it being a doped material, Ni K-edge *homogeneously* probes single Ni atoms in the 1T-MoS_2_ structure, while Mo K-edge *heterogeneously* probes all Mo atoms irrespective of their proximity to a Ni dopant. This is an inherent difficulty in measuring the host lattice atoms in doped and composite materials. Despite this difficulty, the Mo K-edge and Ni K-edge data do appear to correlate. We have revised the manuscript to include this information (Line 220-227, Page 13).

*(v) During fitting of Ni K edge FT [χ(k)k3] vs. R data also Ni-Mo path has been taken, however in the plots of as-prepared and acidic samples hardly any peak is visible at around 3 Ả. Here also the authors have to establish this with more proof. If it is there why it has not been taken in case of the alkaline sample? It is getting submerged in the large peak of Ni-Ni? More explanation is needed on this.*

**Author Response:** We thank the reviewer for raising this point. According to the reviewer’s comments and other reviewers’ comments, the Ni K-edge fits have been re-performed for the as-prepared dry sample and the sample in acidic conditions. This is a total of four data sets that have been re-analyzed. It is known from the previous work [Huang, Y. et al. *Nature Communications*, **2019**, *10*, 982, Ref. 28] that Ni doping further introduces O doping, resulting in Ni-O moieties in the structure. In the original manuscript, we attempted fitting using variable coordination numbers of first shell Ni-S and Ni-O paths. This was unsuccessful and would result in unrealistic (>6) coordination numbers, so we opted to only include Ni-S paths, which are much larger in intensity relative to O paths due to scatterer size.

We have opted to now re-fit the data with a model including Ni-O first shell scattering with a fixed Ni-O coordination number of 1 based on the elemental analysis data of the previous work. Table 2 and Figure 3 have been updated accordingly. In the dry As-prepared sample, we note that there is some second shell structure at approximately 3 Å. The feature is very weak but is not fit without an included Ni-Mo path. We rationalize the inclusion of the Ni-Mo path because the coordination number of Ni-Mo fits to 0.8 in this particular spectrum and is likely correlated to the presence of an Ni-O moiety because the smaller O ligand of Ni allows some degree of second shell scattering to be observed. This feature shifts significantly to shorter distance in acidic conditions, especially under applied potential.

For the alkaline condition, we observed a significantly different species. We have revised the manuscript and SI further to clarify this point (Page 11, lines 202-204; Page 13, lines 230-231, lines 234-236, and lines 238-241). Upon immersion in alkaline media, Ni atoms become labile in the structure and form clusters associated with the 1T-MoS_2_ lattice. We have added the below figure to the SI (Figure S9) to compare to NiO reference data to better show this structural transformation, and to also show that the species observed is not simply NiO. *Ex situ* XPS (Figure S13 added in this revision, shown below) suggests oxidation of Ni under alkaline conditions but not under acidic conditions. While XANES and XPS suggest a species electronically similar to NiO, the EXAFS show a clearly different species. EXAFS fitting corroborated this result and suggested that the dominant first shell coordinating atom of Ni becomes O after immersion in alkaline electrolyte and with a pre-catalytic potential (0V vs. RHE). We have further revised the manuscript to refer to this pre-catalytic species as NiS_x_O_y_ (Page 13, lines 238-241).

**Figure S9**. Comparison of NiO reference data to Ni@1T-MoS_2_ No Potential, Alkaline data. While the XANES edge (a) and first derivative (b) show similar oxidation state and features, the EXAFS oscillations (c) are significantly different. The R-space spectra were normalized at the first shell scattering feature to yield the comparison in (d) which shows that Ni in Ni@1T-MoS_2_ exhibits significantly longer first shell scattering interactions and, furthermore, shows different second shell scattering interactions. It can be interpreted that the Ni-Ni interactions in Ni@1T-MoS_2_ are much longer in the second shell, implying that the species formed is not simply NiO, but rather a species still associated with 1T-MoS_2_, i.e. NiS_x_O_y_.

**Figure S13.** XPS analysis before and after HER stability tests of Ni@1T-MoS_2_. (a-c) High-resolution XPS signals of (a) Mo3d; (b) Ni2p; (c) S2p for the Ni@1T-MoS_2_/CFP electrocatalyst.

*(vi) The significant change in the FT [χ(k)k3] vs. R plot when a catalytic potential of -0.76 V is applied in alkaline medium, the peak becomes very broad and fitting shows that the Ni-Ni bond length which is otherwise at 3.1Ả becomes 2.53Ả to fit this broad peak. It needs clear explanation. How Ni-Ni peak can get shortened so much?*

**Author Response:** The confusion is that in the No Potential, Alkaline condition and the pre-catalytic 0V vs. RHE condition, which probes the NiS_x_O_y_ species, the Ni-Ni paths are second shell paths. Upon application of a catalytic potential (-0.76V vs. RHE), we observed the significant structure change correlated with the observation of Ni^0^ in XANES, and therefore in this spectrum, the Ni-Ni path is a first shell path. We have revised the manuscript accordingly (Page 14, line 256) and hope that the revisions to the manuscript have made this clearer.

*(vii) I am also very much perplexed about fig. 5 where the authors have claimed that Ni has gone back to Ni-O phase from metallic Ni phase after catalytic reaction. But then why the strong peak around 2.75Ả is appear in the FT [χ(k)k3] vs. R plot of the ex-situ sample which if I have understood correctly is due to Ni-Ni*

**Author Response:** This is because the catalytically active species has Ni-Ni in the first shell whereas the pre-catalytic species (NiS_x_O_y_) has Ni-Ni interactions in the second shell (see response to vi). This is an important point for the reversibility that we see such a strong contrast in the local structure.

We thank the reviewer again for the above insightful points, which have been very helpful to guide us to better explain the observations in this study.

***Reviewer #2***

*This manuscript reports in situ XAS characterizations of Ni single atoms doped on basal plane of 1T-MoS2, and the HER catalytic mechanisms in both acidic and alkaline solutions, especially the role of Ni single atoms were thoroughly investigated. It was found that under acidic conditions the Ni atoms remains stable as its native structure (status) while in alkaline media, a reversible feature of metallic Ni species (Ni0) is observed. The Ni-doped 1T-MoS2 catalyst system has been reported and other reports on similar catalysts also exist, but the value of the current work is the in situ studies to understand the true roles of single atom as the active sites in MoS2 toward HER. Therefore, I recommend it to be published in Nature Comm. However, there are some major issues should be addressed:*

*1. Do you have clear evidence that Mo does not go to Mo (0), as Ni does in the alkaline HER conditions? It sounds like the Mo foil data were collected as reference but you did not specifically compare the EXAFS data. I am asking this because in reality, NiMo alloy has been the benchmark HER catalyst in alkaline conditions.*

**Author Response:** We thank the reviewer for the insightful comments that guide our revision. As suggested by the reviewer, we have now compared the XANES and EXAFS spectra of Mo foil and the samples in the figures below, where it is clearly seen that the oxidation state of Mo does not change or go to Mo^0^ during the course of catalysis in either acidic or alkaline conditions. The EXAFS spectra of all samples remain very similar in structure but vary in oscillation amplitude. This change is explained as changes in coordination number of Mo due to coordination to solvent atoms. We observed this trend in the EXAFS where the fitting does show an overall increased coordination number at Mo under these conditions. Given the fitting uncertainties in coordination number (± 0.5; manuscript further revised to include uncertainty in Table 1 and Table 2), we cannot comment further on exactly what coordination changes occur, however we suggest that there are some surface states that were undercoordinated in the dry as-prepared sample that became coordinated with aquo or hydroxyl ligands in solution. We have revised the manuscript to incorporate the possible reason for the increased coordination number at Mo at Page 10, lines 164-167 in the revised manuscript. We have kept the explanation in the manuscript broad to not suggest specific species that we cannot comment on further.

To further confirm that Mo does not go to Mo (0), X-ray photoelectron spectroscopy (XPS) was applied to analyze the Mo 3d spectra of the Ni@1T-MoS_2_ catalyst before and after HER in both acidic and alkaline solutions for 1 h electrolysis. As shown in Figure S13 below, the high-resolution Mo 3d spectra were manly deconvoluted into two peaks in the original and acid-operated samples. These two characteristic peaks of Mo 3d_5/2_ (~228.5 eV) and Mo 3d_3/2_ (231.6 eV) suggest the dominating oxidation state of Mo is +4 in Ni@1-TMoS_2_ (Nat. Chem. 2018, 10, 638, DOI: 10.1038/s41557-018-0035-6). Interestingly, the Mo 3d peaks in basic condition shows a slight shift of ~0.3 eV to higher binding energy compared to those of original and acidity ones. Moreover, a higher Mo^6+^ peak is generated after electrolysis under basic condition, indicating that Ni@1T-MoS_2_ catalyst probably undergoes a surface oxidation in the electrolysis process. Combining XAS and XPS results, we believe that Mo does not go to Mo (0) in HER. However, under prolonged basic-condition electrolysis, Mo will gradually undergo surface oxidation. Discussion of these XPS characterizations have been incorporated into the main text (Pages 18-19, lines 303-316).

**Figure R1.** Top panels are EXAFS (left) and XANES (right) comparisons of the Mo Foil reference data to the As-Prepared sample. Bottom panels include all Mo K-edge data sets in this work.

**Figure S13.** XPS analysis before and after HER stability tests of Ni@1T-MoS_2_. (a-c) High-resolution XPS signals of (a) Mo3d; (b) Ni2p; (c) S2p for the Ni@1T-MoS_2_/CFP electrocatalyst.

*2. The authors have not considered the defects and vacancies situations in their solution synthesized/converted 1T-MoS_2_ (which are prone to have defects). These previous reports (J. Am. Chem. Soc. 2016, 138, 7965, J. Am. Chem. Soc. 2016, 138, 16632, the cited ref. 5) showed that vacancy defects are very important to catalytic activity. Could it be possible that some current data already reveal something about the defects that were not paid attention to? If vacancies were very prevalent around the Mo metal, I think we would see a <6 coordination number from the fitting. The authors do see a <6 CN in the first-shell fitting for Mo, but they say there is an additional Mo-O path that is convoluted and hard to fit.*

**Author Response:** We thank the reviewer for raising this point regarding the coordination number of Mo. We do believe that the Mo sites contain defects as prepared. Building on the response to Reviewer Question number 1, the increased coordination observed in the aqueous electrolytes in the absence of any other observed structure changes is evident of Mo sites with lower than expected coordination number. In combination with the fitting uncertainties now reported in coordination number of ± 0.5, we do expect there to be Mo-O paths in the structure, but these are difficult to account for because of their size relative to S, and because they are associated with Ni dopant sites, meaning that not every Mo site is expected to have Mo-O coordination based on our previous work [Huang, Y. et al. *Nature Communications*, **2019**, *10*, 982]. Based on this logic, we have further reanalyzed some of the Ni K-edge data to account for these Ni-O interactions (See further responses to Reviewer Question 4 where this reanalysis is discussed in full detail). As mentioned by the reviewer, vacancy defects have shown to be active in 1T-MoS_2_ (J. Am. Chem. Soc. 2016, 138, 7965, J. Am. Chem. Soc. 2016, 138, 16632, the cited ref. 5), it seems surprising that in this work, very little change was observed at the Mo K-edge. However, given the significant change in catalytic properties due to Ni doping (Figure S4), it is understandable why the Ni site is observed to be the active site in this work. We have slightly revised the applicable comments in the main text (Page 10, lines 170-174). Regarding undoped samples, it might be interesting to develop synthetic methods that can be tuned to systematically introduce defects and measure the impact of the defect states on the structure using EXAFS, which however is outside of the scope of this work.

**Figure S4**. Linear Sweep voltammetry of Ni@1T-MoS_2_ under acidic 0.5 M H_2_SO_4_ (a) and alkaline 1M NaOH (b) conditions.

*3. The authors do not seem to adequately explain why the Ni is dark and the Mo are bright, when they explicitly point out that literature demonstrates the opposite. It seems like electrons should more easily pass through the low Z element. The zone axis is not stated. It seems the cited papers also support that this trend should be opposite. Could these be vacancy sites instead? A zoom out TEM image should also be shown to demonstrate the sample region. Does the density of nickel active sites make a difference, and should this be further characterized? No attempt at characterizing the density of nickel active sites of amount of nickel in the structure appears to be made. Figure S10 and the implications of when Ni atoms become less isolated should be mentioned in the text along with Figure 2.*

**Author Response:** We thank the reviewer’s suggestions. Figures 2 and S2 are annular dark-field (ADF) STEM images, which are sensitive to electrons scattering. As a result, the intensity of ADF signal is proportional to the atomic number (*Z*) of observed materials and scales as *Z*^1.6~2^ [Nature 2010, 464, (7288), 571-574]. Therefore, the STEM intensity of single doped Ni atom (*Z*_Ni_ = 28) should be dimmer than that of Mo atom (*Z*_Mo_ = 42) in MoS_2_ lattice. The simulated spectrum intensity matches with the experimental intensity, indicating that the bright spots are Mo and dark spots are Ni, rather than a vacant site in STEM images. This explanation has now been added to the revised manuscript (Page 5, lines 99-104).

**Figure S2.** HAADF-STEM characterization of Ni@1T-MoS_2_ STEM with the experimental and simulated spectra, which indicates the bright spot is Mo and dark spot is Ni rather than a structural vacant site.

*4. Since the oxygen element is involved in precursor, the influence (contribution) from oxygen for the properties can’t be completely excluded and in fact, the authors have claimed that although no Mo-O scattering in first shell was observed, ICP analysis with a higher resolution demonstrated the oxygen doping in MoS2. Please provide more discussion on this.*

**Author Response**: We thank the reviewer for this valuable comments. According to the reviewer’s comments and the results from our previous work [Huang, Y. et al. *Nature Communications*, **2019**, *10*, 982] that Ni doping further introduces O doping, resulting in Ni-O moieties in the structure, we re-performed the Ni K-edge fits for the as-prepared dry sample and the sample in acidic conditions to include the contribution from O to the structure of the sample. This is a total of four data sets that have been re-analyzed. In the original manuscript, we attempted fitting using variable coordination numbers of first shell Ni-S and Ni-O paths. This was unsuccessful and would result in unrealistic (>6) coordination numbers, so we opted to only include Ni-S paths, which were much larger in intensity relative to O paths due to scatterer size. We have now opted to re-fit the data with a model including Ni-O first shell scattering with a fixed Ni-O coordination number of 1. While the incorporation of these moieties in the structure does result in Mo-O bonds, those are only present when Mo is directly adjacent to a Ni atom. Due to it being a doped material, Ni K-edge *homogeneously* probes single Ni atoms in the 1T-MoS_2_ structure, while Mo K-edge *heterogeneously* probes all Mo atoms irrespective of their proximity to a Ni dopant. We have added this point at Page 13, lines 225-227 in the revised manuscript. This is an inherent difficulty in measuring the host lattice atoms in doped and composite materials. Therefore, no Mo-O first shell paths are included because those paths are only represented in measurable amounts in the Ni K-edge data. The discussion based on these new analyzed results are added in Page 13, lines 219-225 in the revised manuscript. In terms of the influence of O doping on the HER performance, since we have discussed the O co-dopant effect in the previous paper [Huang, Y. et al. *Nature Communications*, **2019**, *10*, 982], we did not discuss this topic in this paper. Here, we cited the previous descriptions and figures as the following:

“The NiO@1T-MoS_2_/CFP catalysts with different oxygen incorporations were obtained by controlling the synthesis temperature. In this work, various synthesis temperatures (160, 180, 200, and 220 °C) were used to control the sulfuration process of NiO@1T-MoS_2_/CFP, forming T-160, T-180, T-200 and T-220, respectively. As the sulfuration process becomes less sufficient with decreasing reaction temperature, an increase amount of oxygen incorporated into the original Ni–O or Mo–O bonds occurs. Thus, changing the temperature used during synthesis clearly impacts catalytic activity and HER performance. As shown in Supplementary Figs. [15](https://www.nature.com/articles/s41467-019-08877-9#MOESM1), the four samples studied (T-160, T-180, T-200, and T-220) all exhibit similar nanosheet structures. Raman spectra of these samples to investigate their structural information is provided in Supplementary Figs. [16a](https://www.nature.com/articles/s41467-019-08877-9#MOESM1). As the synthetic temperature starting at 200 °C increases, a weak characteristic Raman peak at 406 cm^−1^ (gray area in Supplementary FigS. [16a](https://www.nature.com/articles/s41467-019-08877-9#MOESM1)) associated with the A1g first-order phonon modes of 2H phase emerges. Further assessment of the temperature-varied samples in 1.0 M KOH electrolyte conducted showed that the T-180 sample exhibits the lowest overpotential (65 mV for 20 mA cm^−2^ current density) of all the samples tested. Results are shown in Fig. [5e](https://www.nature.com/articles/s41467-019-08877-9#Fig5). This result suggests a volcano-like relationship between overpotential and the oxygen incorporation content (inset in Fig. [5e](https://www.nature.com/articles/s41467-019-08877-9#Fig5)). The corresponding Tafel plots (Fig. [5f](https://www.nature.com/articles/s41467-019-08877-9#Fig5)) indicate that the water dissociation step kinetics are effectively facilitated for T-180 (52 mV dec^−1^). The inferior HER performances of T-200 and T-220 are most likely due to the emergence of 2H-MoS_2_ phase witnessed in Raman analysis of the samples at higher temperatures (Supplementary Fig. [16a](https://www.nature.com/articles/s41467-019-08877-9#MOESM1)). T-160's lower HER activity is likely due to the sulfuration process being less efficient at 160 °C, which is demonstrated by the higher oxygen content present upon EDX analysis (Supplementary FigS. [16b](https://www.nature.com/articles/s41467-019-08877-9#MOESM1)) and elemental analysis comparison with T-180 (Supplementary Table [1](https://www.nature.com/articles/s41467-019-08877-9#MOESM1)). The exact content of oxygen atoms in the samples obtained via elemental analysis are summarized in Supplementary Table [1](https://www.nature.com/articles/s41467-019-08877-9#MOESM1). Results reveal that the oxygen content varies from 6.38 to 1.35% with an increase in temperature. It should be noted that all four samples were exposed to air at similar conditions before elemental analysis, thus suggesting possible oxygen incorporation (incomplete sulfuration) in NiO@1T-MoS_2_ outside of surface oxidation by air.”

**Figure S15 from Ref 28.** The TEM images of NiO@1T-MoS_2_ nanosheets synthesized at different temperatures, including **a**. T-160. **b**. T-180. **c**. T-200. **d**. T-220.

**Figure S16 from Ref 28.** **a**. The Raman spectra of NiO@1T-MoS_2_ nanosheets (denoted as T-n, where n denotes the synthesis temperature in ^o^C) synthesized at different temperatures. **b**. EDS results showing oxygen contents of different T-n samples. The accurate oxygen contents are summarized in Table S1.

**Figure 5 from Ref.28 | HER catalytic performance and EIS responses for** **NiO@1T-MoS_2_.** **a**. Polarization curve of all catalysts in 1.0 M KOH (scan rate: 5 mV s^-1^ under a three-electrode configuration). **b**. Tafel plots. **c**. Electrochemical impedance spectroscopy (EIS) of NiO@1T-MoS_2_ at various overpotentials. **d**. Stability tests of NiO@1T-MoS_2_ (inset: the long-term durability tests at η = 130 mV for 30 hours). **e**. Polarization curves (inset: overpotentials to drive 20 mA cm^-2^). **f**. The corresponding Tafel slopes of Ni@1T-MoS_2_ synthesized at various temperatures: 160°C (T-160), 180°C (T-180), 200°C (T-200), and 220°C (T-220) samples.

**Table S1 from Ref 28.** Summary of elemental analysis of varied XO@1T-MoS_2_ electrocatalysts

| XO@1T-MoS_2_  Catalysts | Mo content (wt. %)^[a]^ | S content (wt. %)^[a]^ | Mo/S Atomic ratio | X=Fe content (wt. %)^[a]^ | X=Co content (wt. %)^[a]^ | X=Ni content (wt. %)^[a]^ | X/Mo Atomic ratio | N content (wt. %)^[b]^ | O content (wt. %)^[c]^ |
| --- | --- | --- | --- | --- | --- | --- | --- | --- | --- |
| 1T-MoS_2_ | 50.87 | 35.48 | 1:2.09 | - | - | - |  | 6.03 | 3.61 |
| FeO@1T-MoS_2_ | 45.98 | 34.95 | 1:2.27 | 4.51 | - | - | 1:5.93 | 7.27 | 4.81 |
| CoO@1T-MoS_2_ | 44.29 | 35.37 | 1:2.39 | - | 4.58 | - | 1:5.94 | 7.55 | 4.52 |
| NiO@1T-MoS_2_ (T-180) | 43.85 | 34.49 | 1:2.35 | - | - | 4.50 | 1:5.96 | 7.34 | 4.59 |
| NiO@1T-MoS_2_ (T-160) | 41.98 | 34.20 | 1:2.44 | - | - | 4.41 | 1:5.83 | 8.63 | 6.38 |
| NiO@1T-MoS_2_ (T-200) | 46.90 | 35.13 | 1:2.24 | - | - | 4.68 | 1:6.13 | 5.47 | 2.52 |
| NiO@1T-MoS_2_ (T-220) | 49.76 | 35.61 | 1:2.14 | - | - | 4.99 | 1:6.10 | 2.41 | 1.35 |

[a] Data are determined by ICP-AES elemental analysis; [b] Data are determined from C, H and N elemental analysis; [c] Data are calculated from results of [a] and [b].

*5. Are the XRD and Raman results shown in Figure S2c and f shown for the same NiO@1T-MoS2 catalyst grown directly on CFP or is this a powder sample? There’s no apparent peak in the XRD as a result of the CFP, and the crystallographic labels for MoS2 are not included, and the corresponding characterization of the 1T-MoS2 catalyst is also not included.*

**Response**: The XRD and Raman results shown in Figure S2c and f are from powder Ni@1T-MoS_2_ samples without CFP. The standard XRD peaks of MoS_2_ (PDF#75-1539) and the XRD and Raman characterizations of the 1T-MoS_2_ catalyst and the corresponding discussion of these results have also been included in the revised Figure S1 and SI, respectively. Note that the figure numbering has been changed to Figure S1 in the revised manuscript.

X-ray diffraction (XRD) analysis was performed to examine the crystalline structure of Ni@1T-MoS_2_ electrocatalyst (Figure S1c). The XRD of Ni@1T-MoS_2_ showed clear characteristic diffraction peaks at around 10.35°, 32.84°, and 35.70° (red plot in Figure S2c), corresponding to the (002), (100), and (102) planes of hexagonal MoS_2_ (PDF#75-1539). However, the peak of Ni@1T-MoS_2_ at 2θ = 10.25° represents a stacked, multilayered structure, which is lower than that of MoS_2_ (PDF#75-1539) at 14.13°, indicating that this multilayered spacing of 1T-MoS_2_ is larger than that of the bulk 2H-MoS_2_ (6.3 Å, PDF#75-1539). Previous publications also verified analogous XRD features for 1T-MoS_2_ or few-layered MoS_2_ [*Applied Catalysis B: Environmental* 251 (2019) 87–93; *J. Am. Chem. Soc.* 135 (2013), 10274-10277]. The Raman peaks of Ni@1T-MoS_2_ and 1T-MoS_2_ are almost same, corresponding well with their similar structures. The characteristic Raman peaks at 147, 214, 236, 283, and 335 cm^−1^ (Figure S1f) can be associated with the phonon modes in 1T-MoS_2_ confirms the formation of a pure 1T-MoS_2_ nanosheet. These discussions have been added in the main text, pages 4-5, lines 80-91.

**Figure S1.** Structure characterizations. (a) SEM image of Ni@1T-MoS_2_, scale bar: 200 nm (inset: low magnification SEM image, scale bar: 5 μm). (b) TEM image of Ni@1T-MoS_2_ (scale bar: 100 nm). (c) The XRD patterns of the Ni@1T-MoS_2_ and 1T-MoS_2_ nanosheets. (d) and (e) EDX mappings of Ni@1T-MoS_2_ (scale bar: 100 nm). (f) Raman spectra of NiO@1T-MoS_2_ and 1T-MoS_2_ nanosheets.

*6. The indicated “shift” in the first shell in acidic media in Figure 3c seems fairly minute – seemingly only barely outside of the error of the fitting according to Table 1? Perhaps the values from the fits could be somehow included in the figure to more effectively communicate the changes, as they are difficult to observe from the data.*

**Author Response:** We thank the reviewer for carefully reading our manuscript. We agree with the reviewer that the shifts are small. In general, the uncertainty in resolvable distance in the first shell is ± 0.02 Å, sometimes reported as low as ± 0.01 Å for some analysis routines and data sets. Shifts of that magnitude are difficult to observe by eye and it is more common to make the necessary comparisons quantitatively. The shifts observed in this data are between 0.03 – 0.05 Å and, while being reasonably different to compare quantitatively, are difficult to observe in the data qualitatively. In addition, it would be difficult to include the values onto the figure without overcrowding the figure. Therefore, we used the vertical dotted line to show the shifts in the figure and listed the more definite quantitative data in Table 1.

*7. The discussion of 1T to 2H transformation could be more convincing with before and after ex-situ XRD or SAED patterns.*

**Author Response:** We have tried to characterize the XRD of Ni@1T-MoS_2_/CFP (CFP=carbon fiber paper) before and after electrolysis, but the signal of Ni@1T-MoS_2_ is not observable in either case due to sensitivity limitations. However, the Raman spectra of Ni@1T-MoS_2_/CFP support that the phase of 1T-MoS_2_ was maintained in the electrolysis process. The ex-situ Raman spectra of Ni@1T-MoS_2_/CFP before and after HER (Figure S14) show that Ni@1T-MoS_2_ retains its morphology and 1T-phase structure after long-term HER stability testing (also reported in Ref. 28). This is in good agreement with EXAFS results, where a 1T to 2H phase transition is not observed, supporting that the active catalyst remains in the 1T phase. The results from Raman spectra have been added into Figure S14 and a corresponding brief discussion on these results is added into Page 19 line 316-321 in the revised manuscript.

**Figure S14.** Raman characterization of NiO@1T-MoS_2_/CFP after long-term HER stability testing in basic electrolyte. No significant changes are observed and are consistent with the peaks in Figure S2.

*8. The conclusions derived from Figure 4 and 5 make sense but lead to the questions about the absence of a major Ni-Mo scattering path in the pristine sample. The authors state that an equivocal Mo-Ni scattering path doesn’t occur under applied potential but given that the Ni atoms are sparsely dispersed across the sample, this being an intense enough scattering pathway to observe for Mo seems unlikely anyways. If Mo-Mo and Ni-Mo is always too weak to be observed, which seems like the case, perhaps Ni-Mo shouldn’t be attempted to fit in the acidic Ni K-edge results.*

**Author Response:** We thank the reviewer for raising this point. As in Question 4, the Ni K-edge fits have been re-performed for the as-prepared dry sample and the sample in acidic conditions with a fixed first shell Ni-O coordination number of 1. In the dry as-prepared sample, we note that there is some second shell structure at approximately 3 Å. The feature is very weak but is not fit without an included Ni-Mo path. We rationalize the inclusion of the Ni-Mo path because the coordination number of Ni-Mo fits to 0.8 in this particular spectrum and is likely correlated to the presence of an Ni-O moiety because the smaller O ligand of Ni allows some degree of second shell scattering to be observed. This feature shifts significantly to shorter distance in acidic conditions, especially under applied potential and is required for an adequate fit.

*9. Both of the cited examples of metal lability in graphene are under electron irradiation. A more relevant example of surface reconstruction could be more helpful?*

**Author Response:** Based on the reviewers’ comments, we have changed the references to be more relevant to surface reconstruction (Nakatsu et al. *Phys. Chem. Chem. Phys.* 2017, 19, 4967−4974; Li et al. *ACS Catal.* 2019, 9, 2521−2531; Lasalle-Kaiser et al., J. Am. Chem. Soc., 2015, 137, 314-321 (Ref. 13)).

*10. In Figure S4, the long-term stability for Ni@1T-MoS2 seems not very good. If this indicated the instability of Ni atoms in MoS2 matrix during the HER test in both electrolytes? If the Ni single atoms will migrate during the HER test? If it is possible, I think there may be some difference in in situ XAS spectra measurement as a function of the test time.*

**Response**: Based on our post electrolysis XPS experiment and ICP-MS results, we believe that Ni@1T-MoS_2_ under acidic solution is quite stable. However, the same catalyst under alkaline condition is not as stable. The Mo 3d, S 2p and Ni 2p peaks all shifted to higher binding energies, indicating the catalyst is oxidized. With the ICP-MS results (Table S3), we can see that Mo element’s concentration (μg/mL) in the solution increased after electrolysis, but no Ni leached from the catalyst. Despite this loss of Mo, a change in the bulk structure via Raman spectroscopy was not observed and local structure changes were not observed in EXAFS experiments. Changes were not observed in EXAFS, which is likely due to the oxidized Mo species being immediately dissolved in the electrolyte in low concentration relative to the probed material.

Regarding the Ni sites, which were not observed to leach, we do see evidence of Ni oxidation in XPS correlating to the changes observed in EXAFS. Upon immersion in alkaline media, Ni atoms become labile in the structure and form clusters associated with the 1T-MoS_2_ lattice. We have added the figure below to the SI (Figure S9), which is compared to NiO reference data to better show this structural transformation, and to also show that the species observed is not simply NiO. While XANES and XPS suggest a species electronically similar to NiO, the EXAFS show a clearly different species. EXAFS fitting corroborated this result and suggested that the dominant first shell coordinating atom of Ni becomes O after immersion in alkaline electrolyte and with a pre-catalytic potential (0V vs. RHE). We have further revised the manuscript to refer to this pre-catalytic species as NiS_x_O_y_ (Page 13, Lines 238-241). The *ex situ* data show that this NiS_x_O_y_ species remains after catalysis and Ni atoms do not re-disperse in the 1T-MoS_2_ lattice as they were in the as-prepared sample.

**Figure S4.** HER stability tests of NiO@1TMoS_2_ electrocatalyst. (a) the long-term durability tests in 0.5 M H_2_SO_4_ at η = 0.76 V for 1 h. (b) the long-term durability tests in 1.0 M KOH at η = 0.76 V for 1 h.

**Table S3.** The ICP-MS results of the electrolytes before and after HER stability tests of Ni@1TMoS_2_ electrocatalyst.

| Elements contents  (*μ*g/mL) | Before HER stability tests in 0.5 M H_2_SO_4_ | After HER stability tests in 0.5 M H_2_SO_4_ | Before HER stability tests in 1.0 M KOH | After HER stability tests in 1.0 M KOH |
| --- | --- | --- | --- | --- |
| Mo | <0.1 | <0.1 | <0.1 | 0.4713 |
| Ni | <0.1 | <0.1 | <0.1 | <0.1 |

**Figure S13.** XPS analysis before and after HER stability tests of Ni@1T-MoS_2_. (a-c) High-resolution XPS signals of (a) Mo3d; (b) Ni2p; (c) S2p for the Ni@1T-MoS_2_/CFP electrocatalyst.

**Figure S9**. Comparison of NiO reference data to Ni@1T-MoS_2_ No Potential, Alkaline data. While the XANES edge (a) and first derivative (b) show similar oxidation state and features, the EXAFS oscillations (c) are significantly different. The R-space spectra were normalized at the first shell scattering feature to yield the comparison in (d) which shows that Ni in Ni@1T-MoS_2_ exhibits significantly longer first shell scattering interactions and, furthermore, shows different second shell scattering interactions. It can be interpreted that the Ni-Ni interactions in Ni@1T-MoS_2_ are much longer in the second shell, implying that the species formed is not simply NiO, but rather a species still associated with 1T-MoS_2_, i.e. NiS_x_O_y_.

*11. The author claimed the reversible feature of Ni single atom in alkaline conditions can be observed from immersion in electrolyte and the application of a catalytic potential. If this behavior could be well repeated after a long-term test.*

**Author Response:** We thank the reviewer for the suggestion. After immersion in electrolyte, the native Ni species in as-prepared Ni@1T-MoS_2_ is permanently converted to the NiS_x_O_y_ species (see Reviewer Question 10 response). This species then reversibly forms the Ni^0^ active species under alkaline conditions at an applied catalytic potential. The *ex situ* EXAFS data in this work shows that the Ni and Mo sites have similar local structure after catalysis, however XPS and ICP-MS showed Mo oxidation and leaching, respectively.

Based on these results, we believe that it would be interesting to perform a set of long EXAFS experiments in which the material is poised at a catalytic potential, and then the material is allowed to re-oxidize to NiS_x_O_y_ species, and this process is repeated several times to investigate exactly how this reversibility changes from several cycles. This could very well be the focus of future work, but is outside the scope of this study. When this work is published, however, it would be easier to justify the Beam Time in a User Proposal to perform such an experiment. The experiment would also need to be performed at Mo K-edge. It would further be interesting to investigate the local structure changes at a broader range of pH values.

*12. Other examples of TM-atom doped MoS2 catalysts: ACS Energy Lett., 2018, 3, 2685. DOI: 10.1021/acsenergylett.8b01567. This one specifically compares the acidic vs. alkaline HER on MoSe2 and MoS2: ACS Energy Lett., 2017, 2 , 2234.*

**Author Response:** We thank the reviewer for suggesting these highly applicable references and have accordingly cited them in the main text on Page 16.

*13. Ref. 28 and 30 are duplicates. In general, there could be more careful reference citation practice in general.*

**Author Response:** We have revised the manuscript and change the citations as the reviewer points out.

We highly appreciate the reviewer’ comments that guide our revision and hope that, with these revisions, the manuscript has improved to meet the criteria of Nature Communication.

***Reviewer #3****:*

*The paper by Patengale et al. describes in situ and operando X-ray absorption studies, which aim is to demonstrate the contribution a Ni single atome electrocatalyst on T-MoS2 for hydrogen evolution. Although the topic is of interest and the use of in situ XAS is appropriate, the conclusions are not supported by the data collected. More specifically, the collected data are not analysed in sufficient depth to allow drawing such conclusions. As a consequence, I have to reject this paper from Nature communications. Besides, I am not convinced that a communication is the best format for such a paper, which would benefit from a more thorough analysis and discussion of the data collected.
Here are several points that justify my decision and could help authors improve their paper:*

[Authors have numbered the points below for clarity]
 *1.) In the abstract and introduction, the term “native environment” is used twice to describe the structure of the nickel ion under standard catalytic conditions in acidic conditions. This is a bad start, since this vague term just suggests that the authors cannot describe precisely what this environment exactly is.*

**Author Response:** We highly appreciate the reviewer’ insightful comments and suggestions that guide our revision. The term “native structure” in this work refers to the local structure of Ni in the “as-prepared” sample. The term “native structure” is synonymous with the combination of qualitative XANES and quantitative EXAFS results obtained for that atom in as-prepared Ni@1T-MoS_2_. Interestingly, we saw very different local structure for the active Ni site in acidic vs basic conditions using *in situ* EXAFS. In order to reduce the confusion, we have now changed the word “native” to “local” in the abstract and to “intrinsic” throughout the manuscript (Page 13, lines 227 and 231; Page 20 line 348).

*2.) The synthesis description mentions the synthesis of POMs with Fe, Co or Ni as hetero-anion, but only the Ni-substituted one is studied in the paper. Why mention the other metals? Also, it would be very important to synthesise the same POM without any (deliberate) addition of nickel, in order to assess the actual role of nickel in catalyst and in the spectroscopy.*

**Author Response**: We apologize for the mistake that we have made and thank the reviewer for bringing this to our attention. The Ni POM synthesized procedure was revised accordingly with the addition of 1T-MoS_2_ as control sample:

“The synthesis of Ni@1T-MoS_2_ HER electrocatalyst follows the previously published protocols.^28^ The synthesis procedure was conducted as follows. First, a Anderson-type POM nanocluster precursors, [(NH_4_)_4_[NiH_6_Mo_6_O_24_]·5H_2_O (NiMo_6_) were prepared. It contains well-defined, 1:6 heteropolyanion clusters, composed of a single metal heteroatom NiO_6_ octahedron with six edge-sharing MoO_6_ octahedrons. The as-prepared NiMo_6_ precursors will react with thioacetamide at 180°C for 24 h in the presence of carbon fiber paper (CFP, 1 × 2 cm^2^) to give rise to the corresponding Ni@1T-MoS_2_/CFP electrocatalyst. The loading amount of Ni@1T-MoS_2_ on CFP is about 1mg cm^−2^. The facile sulfuration reaction incompletely replaces O atoms with S atoms and generates 1T-MoS_2_ nanosheets randomly decorated with Ni single atoms (Figure 1). ^28^”

All chemicals were obtained as reagent grade chemicals from Alfa Aesar® unless noted.

*Preparation of NiMo6 precursor*: The (NH4)_4_[NiH_6_Mo_6_O_24_]·5H_2_O (NiMo6) precursor was prepared according to a modified published procedure. (NH_4_)_6_Mo_7_O_24_·4H_2_O (denoted Mo7, 5.19 g, 4.2 mmol, 99%) was dissolved in DI water (80mL) and then heated to 100°C. Ni(NO_3_)_2_·6H_2_O (1.16 g, 4mmol, 99.99%) was dissolved in water (20mL), which was added to the above solution with stirring. The mixture was kept heating and stirring to give rise to a deep green solution. The crude product (5.4 g) was isolated with evaporation and filteration. The green targeted product (4.6 g, 79.1% yield based on Mo) was obtained by recrystallization in hot water (80 °C) two times, then dried under vacuum. Elemental analysis calcd (%) for H_32_N_4_O_29_NiMo_6_ (M=1186.60 gmol−1): H, 2.72; N, 4.72; Mo, 48.51; Found: H, 2.70; N, 4.66; Mo, 48.62. IR (KBr pellet, major absorbances, cm^−1^): 3402 (νasOH, m), 3152 (νasNH, m), 1627 (δOH, m), 1402 (δNH, s), 929 (νMo=O, vs), 876 (νMo=O, vs), 635 (νMo-O-Mo, vs), 577 (νNi-O-Mo, w).

*Preparation of Ni@1T-MoS_2_/CFP* : The Ni@1T-MoS_2_ catalyst was prepared according to our previous work.^28^ The as-prepared NiMo6 (50mg, 0.042mmol) precursors, thioacetamide (TAA, 80mg, 1.065 mmol, 98%) and CFP (1 × 2 cm^2^, Toray carbon paper, TGP-H-60) were mixed in 10mL H_2_O, transferred into a 20 mL Teflon autoclave, and heated at 180°C for 24 h to give rise to the corresponding Ni@1T-MoS_2_/CFP electrocatalyst. The loading amount of Ni@1T-MoS_2_ on CFP is about 1mg cm^−2^. (Note: CFP represents conductive carbon fiber paper, which can serve as substrate to enable the loading of Ni@1T-MoS_2_ catalyst, forming Ni@1T-MoS_2_/CFP).

*Preparation of 1T-MoS_2_* : 80 mg thioacetamide and 50 mg (NH_4_)_6_Mo_7_O_24_·4H_2_O (Alfa Aesar) were dissolved in 10 mL DI water under sonication treatment for 20 min to form homogeneous solution. Afterwards the solution was transferred into a 25 mL Teflon autoclave, and CFP (1 * 2 cm^2^) was placed onto the bottom. The Teflon autoclave was heated at 180 ^o^C for 24 h to give rise to the in-situ growth of 1T-MoS_2_ on CFP substrate. In addition to the 1T-MoS_2_ grown on the CFP, the remained free 1T-MoS_2_ was collected by centrifugation and then was washed with DI water, ethanol and acetone (each for two times). The purified 1T-MoS_2_ was dried in thermal oven for overnight and ground into fine powders. Loading amount of 1T-MoS_2_ on CFP is quantified by 0.5 mg/cm^2^ by comparing the mass difference before and after hydrothermal growth.

**Figure S1.** Structure characterizations. (a) SEM image of Ni@1T-MoS_2_, scale bar: 200 nm (inset: low magnification SEM image, scale bar: 5 μm). (b) TEM image of Ni@1T-MoS_2_ (scale bar: 100 nm). (c) The XRD patterns of the Ni@1T-MoS_2_ and 1T-MoS_2_ nanosheets. (d) and (e) EDX mappings of Ni@1T-MoS_2_ (scale bar: 100 nm). (f) Raman spectra of Ni@1T-MoS_2_ and 1T-MoS_2_ nanosheets.

*3.) Line 74-75, we understand that the material has been deposited on a carbon paper, but there is absolutely no experimental details on this procedure. It need to be provided, at least in the SI.*

**Author Response**: As suggested by the reviewer, we have added the experiment details of how Ni@MoS_2_ was in-situ grown on a carbon paper in the experimental section (Page 22, lines 373-379):

*Preparation of Ni@1T-MoS_2_/CFP*. The Ni@1T-MoS_2_ catalyst was prepared according to our previous work.^28^ The as-prepared NiMo6 (50mg, 0.042mmol) precursors, thioacetamide (TAA, 80mg, 1.065 mmol, 98%) and CFP (1 × 2 cm^2^, Toray carbon paper, TGP-H-60) were mixed in 10mL H_2_O, transferred into a 20 mL Teflon autoclave, and heated at 180°C for 24 h to give rise to the corresponding Ni@1T-MoS_2_/CFP electrocatalyst. The loading amount of Ni@1T-MoS_2_ on CFP is about 1mg cm^−2^. (Note: CFP represents conductive carbon fiber paper, which can serve as substrate to enable the loading of Ni@1T-MoS_2_ catalyst, forming Ni@1T-MoS_2_/CFP.

*4.) Line 77: the word “inevitably” is incorrectly used, as often throughout the aper and suggests that the authors use characterization techniques to demonstrate something that they have already concluded about.*

**Author Response:** We thank the reviewer for finding this issue and we have deleted the word where it appeared in the manuscript.

*5.) Line 86-87: it is assumed that the lower intensity observed on one spot of the STEM image is a clear proof of Nickel insertion, but there might be other explanations for this lower intensity, such as a simple defect in the MoS2 network. The authors should comment about this possibility.*

**Author Response:** Figures 2 and S2 are both annular dark-field (ADF) STEM images, which are sensitive to electrons scattering. As a result, the intensity of ADF signal is proportional to the atomic number (*Z*) of observed materials and scales as *Z*^1.6~2^ [Nature 2010, 464, (7288), 571-574]. Therefore, the STEM intensity of single doped Ni atom (*Z*_Ni_ = 28) should be dimmer than that of Mo atom (*Z*_Mo_ = 42) in MoS_2_ lattice. The simulated spectrum intensity matches with the experimental intensity, indicating that the bright spots are Mo and dark spots are Ni, rather than a vacant site in STEM images. We have added these further explanations to the main text (Pages 5-6, lines 99-108) and have added Figure S2 to support the interpretation. We thank the reviewer for raising this point.

**Figure S2.** HAADF-STEM characterization of Ni@1T-MoS_2_ STEM with the experimental and simulated spectra, which indicates the bright spot is Mo and dark spot is Ni rather than a structural vacant site.

*6.) The paragraph between lines 88 and 95 mentions the electrochemical performances of the Ni@T-MoS2 material with respect to T-MoS2. How was the reference T-MoS2 prepared? Was it prepared and studied by the authors or are the data collected from another work? This is not clear in the current version of the paper.*

**Response**: We apologize for the confusion. Our previous work (see Ref. 28) has discussed the electrochemical performances of the Ni@T-MoS_2_ material with respect to 1T-MoS_2_. Now in order to clarify the Ni@1T-MoS_2_ structure, we have added 1T-MoS_2_ as a control sample in XRD and Raman spectroscopy in the Figure S1, where the discussion on these results are added to line 80-89, page 5.

**Figure S1.** Structure characterizations. (a) SEM image of Ni@1T-MoS_2_, scale bar: 200 nm (inset: low magnification SEM image, scale bar: 5 μm). (b) TEM image of Ni@1T-MoS_2_ (scale bar: 100 nm). (c) The XRD patterns of the Ni@1T-MoS_2_ and 1T-MoS_2_ nanosheets. (d) and (e) EDX mappings of Ni@1T-MoS_2_ (scale bar: 100 nm). (f) Raman spectra of Ni@1T-MoS_2_ and 1T-MoS_2_ nanosheets.

*7.) I am a bit skeptical about the data collected under in situ conditions. The setup described by the authors with a kapton membrane facing the working electrode is too rudimental to be completely confident in the data collected. The amount of nickel in the sample is supposed to be very low, so any contamination from adventitious nickel (in the glue, in the plastic or anywhere else) could compromise the quality and hence the significance of the data. The authors should at least provide blank XAS spectra of a setup where everything is equal, except for the nickel substitution in the 1T-MoS2. The carbon paper itself can often contain metallic traces. Ideally, another setup with the working electrode facing the incident beam should be used. There are plenty of examples of such setup, such as described in King et al. J. Phys. Chem. C 2019, 123, 28533−28549. There are plenty of other papers using electrochemical cells for in situ X-ray absorption that are much more evolved (yet not that complicated) than the one used here.*

**Author Response:** We agree that our setup is simple but is just as appropriate as other designs reported. The cell reported in *King et al. J. Phys. Chem. C 2019, 123, 28533−28549*, as the reviewer cites, is a very nice design. Several components of any cell design may contain or leach metal atoms and it is important to confirm that no background signal is observed from any component. Comparatively, our cell only use glass, Loctite Hysol (which is frequently used in electrochemical applications to secure windows or to insulate wires and electrical connections), and Kapton, which is the most frequently used material on XAS beamlines to prepare powder and pellet samples for measurement. We did not observe interfering background Ni or Mo signal from our cell.

Furthermore, regarding the design, it is well known that X-rays are surface sensitive. In the King’s paper, the X-ray enters the cell from the rear of the electrode, and then fluorescence is detected. Therefore, information on the catalytically active surface states is only obtained if the X-ray penetrates to those surface states. King et al. go through these issues in detail and discuss that low loading is necessary to solve this issue. In our design, the surface faces the X-ray. There is of course the drawback of X-ray attenuation by the solvent, but the electrode is positioned close enough to detect sufficient signal. We are by no means criticizing the King design or other established designs, which are more sophisticated in many ways, but are defending that our design is equally applicable to the experiment performed in this work. It would also be very difficult to use a porous CFP electrode in a sophisticated sandwich cell, because it would either introduce leaks or need to be anchored onto another electrode using a conductive adhesive.

*8.) On figure 3, the XANES is not mandatory, since there is nothing to learn from it. It could be sent to the SI. On figure 3c, there are plain lines and symbols, what do they correspond to? Fits and data? These kind of issues should not be the work of a reviewer but of co-authors! Please proof-read your papers before sending papers for publication!*

**Author Response:** We have chosen to keep the XANES spectra in the main text so that readers do not need to refer to the SI to see XANES for Mo data. We have edited the figure captions to state that the open points are data and the fits are solid lines. We apologize that this was not stated in the original draft. *9.) Line 148: I understand from this sentence that the shortening of the Mo-S bond is due to the fact that nickel is the active in Ni@1T-MoS2. How does that relate? Why would the shortening of an Mo-S bond under catalytic conditions make more sense in a Ni@1T-MoS2 than in amorphous MoSx? This assumption should be explained with sound arguments.*

**Author Response:** We thank the reviewer for the insightful comments that guide our revision. This contraction agrees with structure changes observed at the Ni K-edge further in the manuscript, as Ni-Mo second shell scattering becomes shorter under acidic conditions. Further explanation has been added to Page 13, lines 219-227 in the revised manuscript. Under alkaline conditions, very significant changes in the Ni local structure are observed which likely impact the Mo results.

There exists a layer of difficulty in directly comparing the Ni K-edge results to the Mo K-edge results. Due to it being a doped material, Ni K-edge probes single Ni atoms in the 1T-MoS_2_ structure while Mo K-edge probes all Mo atoms irrespective of their proximity to a Ni dopant. This is an inherent difficulty in measuring the host lattice atoms in doped and composite materials. Despite this difficulty, the Mo K-edge and Ni K-edge data do appear to correlate. We have revised the manuscript to include this point (Page 13, lines 225-227).

We don’t believe that shortening of a Mo-S bond under catalytic conditions in a Ni@1T-MoS_2_ material makes more sense than in an amorphous MoS_x_ and apologize if the statements in that portion of the manuscript were unclear. We were simply making the statement that we did not see pronounced changes at Mo center in Ni@1T-MoS_2_. However, we did see significant changes at the Ni center, owing to it being the catalytic active site. We thank the reviewer for raising this point and have added a clarifying sentence in this portion of the manuscript (Page 10, Line 170-174).

*10.) The analysis of the XANES data presented on figure 4a is a very weak point of the paper. The authors basically claim that the spectra of the as prepared, no potential in acid, 0V and catalytic conditions are identical. Or at least that the changes are so minimal that they don’t mean anything. This is either a poor knowledge of X-ray absorption spectroscopy or a deliberate intention to hide what is not understood in order to orient the results of their study. In both cases, it is a serious problem! The assumption that the species does not change is based on the first derivative of the XANES spectra, which indeed do not show a shift of the main edge position. But XANES is not just about edge position, it can be clearly seen that the spectra are not identical, both from the original spectra and from the derivatives. These differences need to be discussed, even if their origins cannot be clearly identified.*

**Author Response:** We respectively disagree with the reviewer’s statements that we have poor knowledge of X-ray absorption spectroscopy or had any intent to deceive the reader or misrepresent the data, the latter of which is a serious statement. It might be the statements we made about the Mo K edge data with those made about the Ni K edge data (which the reviewer is referring to here), which has introduced misunderstanding. Please see the except below for the related statement to this question. The excerpt begins at Page 10, line 183 in the revised manuscript. For clarity, we have italicized the statements that have been revised and the unrevised statements, originally presented, remain in a Roman typeface (bolded for emphasis):

“**The XANES spectra (Figure 4a) show that significant local structure changes occur throughout the catalysis process**. Beginning from the dry sample, a weak white line feature is observed on-edge (1s-4p transition, 8.350 keV). Such a feature is typically observed for Ni^2+^ with unsaturated coordination and is sensitive to the identity of coordinating atom,^38-41^ suggesting that single-atom Ni preserves an oxidation state of 2+ after replacing Mo atoms. *The oxidation state is further supported by Ni reference spectra (Figure S8).* Moreover, no peak is observed at *2.10* Å in the R-space EXAFS spectra (Figure 4c) where Ni-Ni coordination (first shell) is, correlating well with the previous conclusion that Ni exists as single atom in 1T-MoS_2_. Upon immersing in acidic electrolyte (No potential), it is observed that the shape of the spectrum at above-edge region has little change; however, a shoulder (⁓ 8.340 keV) is observed on the rising part of the edge along with a reduced white line intensity. When the first derivative spectra are compared (Figure 4b), it is apparent that the edge shifts by approximately 3 eV to a lower energy. While this is consistent with reduction of the Ni metal center without applied potential,^42-44^ **structural and electronic factors could contribute and are examined in more detail via EXAFS fitting (*vide infra*).** Further shifting of the edge toward lower energies is observed with the application of pre-catalytic (0 V) and catalytic (-0.76 V) potentials.”

As can be seen from the statements above, we did not make statements that there are no changes in acidic electrolyte. We purposefully highlighted the progression of the XANES spectrum at each condition, citing the appropriate literature references, and explained that the XANES edge changes are due to both structural and electronic factors, the latter of which are further examined via quantitative EXAFS fitting. Note that we have newly added in this revision the statement “The oxidation state is further supported by Ni reference spectra (Figure S8)” and have further corrected the referenced distance to 2.10 Å in order to make the statement clearer. Beyond these revisions, the substantive contents of the above excerpt are unchanged from the as-submitted manuscript.

We should additionally note that the Ni K-edge fits have been re-performed for the as-prepared dry sample and the sample in acidic conditions. This is a total of four data sets that have been re-analyzed according to the reviewer’s comments and other reviewers’ comments. The results in Table 2 and fits in Figure 3 have been updated accordingly in the revised manuscript. It is known from the previous work [Huang, Y. et al. *Nature Communications*, **2019**, *10*, 982] that Ni doping further introduces O doping, resulting in Ni-O moieties in the structure. In the original manuscript, we attempted fitting using variable coordination numbers of first shell Ni-S and Ni-O paths. This was unsuccessful and would result in unrealistic (>6) coordination numbers, so we opted to only include Ni-S paths, which are much larger in intensity relative to O paths due to scatterer size.

We have opted to now re-fit the data with a model including Ni-O first shell scattering with a fixed Ni-O coordination number of 1 based on the elemental analysis data of the previous work. In the dry As-prepared sample, we note that there is some second shell structure at approximately 3 Å. The feature is very weak but is not fit without an included Ni-Mo path. We rationalize the inclusion of the Ni-Mo path because the coordination number of Ni-Mo fits to 0.8 in this particular spectrum and is likely correlated to the presence of an Ni-O moiety because the smaller O ligand of Ni allows some degree of second shell scattering to be observed. This feature shifts significantly to shorter distance in acidic conditions, especially under applied potential. We have further clarified the discussions in Page 13, lines 219-227.

For the alkaline condition, we observe a significantly different species. We have revised the manuscript and SI further to clarify this point (Page 11, lines 202-204). Upon immersion in alkaline media, Ni atoms become labile in the structure and form clusters associated with the 1T-MoS_2_ lattice. We have added the below figure to the SI to compare to NiO reference data to better show this structural transformation, and to also show that the species observed is not simply NiO. While XANES and XPS suggest a species electronically similar to NiO, the EXAFS show a clearly different species. EXAFS fitting corroborated this result and suggested that the dominant first shell coordinating atom of Ni becomes O after immersion in alkaline electrolyte and with a pre-catalytic potential (0V vs. RHE). We have further revised the manuscript to refer to this pre-catalytic species as NiS_x_O_y_ ( Page 13, lines 238-241). We hope that, with these revisions and explanation, we have addressed the reviewer’s concerns and would be happy to answer more questions if it is needed.

**Figure S9**. Comparison of NiO reference data to Ni@1T-MoS_2_ No Potential, Alkaline data. While the XANES edge (a) and first derivative (b) show similar oxidation state and features, the EXAFS oscillations (c) are significantly different. The R-space spectra were normalized at the first shell scattering feature to yield the comparison in (d) which shows that Ni in Ni@1T-MoS_2_ exhibits significantly longer first shell scattering interactions and, furthermore, shows different second shell scattering interactions. It can be interpreted that the Ni-Ni interactions in Ni@1T-MoS_2_ are much longer in the second shell, implying that the species formed is not simply NiO, but rather a species still associated with 1T-MoS_2_, i.e. NiS_x_O_y_.

*11.) One main problem of this study associated with these data is the lack of reference samples. Any good XAS study is based on the comparison with model compounds (unless high level calculations are provided). Why didn’t the author measure and report the XANES and EXAFS spectra of a nickel foil (it is shown in the SI), of NiO, Ni(OH)2, NiS, Ni3S2, NiSO4, the original NiMo POM, etc…This is the basic of any XANES study and I am actually shocked that the authors sent a paper for publication without that, at least in the SI. If reference spectra had been measured, then linear combination fittings could have been performed and a first approximation of the composition of the species could have been obtained. This is true for both the acidic and basic conditions.*

**Author Response:** We thank the reviewer for bringing this problem to our attention and apologize that the reference spectra were not placed in the SI originally. We have now added reference spectra in Figure S8. The NiO data was further compared to the No Potential, Alkaline data in Figure S9. See the response for Question 10 for further explanation of Figure S9.

Linear combination fitting is most useful for determining the contribution of discrete phases in a composite material (for instance, NiO mixed with Ni(OH)_2_; or NiS mixed with Ni_3_S_2_). This is implied in the name of the technique, where the experimental spectrum is a linear combination of two reference spectra. It would also be useful to monitor a reduction or oxidation of a species, with the fully oxidized and reduced reference spectra in-hand. In this case, the Ni center probed is not a linear combination of two discrete species. Keeping this in mind, it still could theoretically provide utility and insight if a specific spectrum contained a linear combination of spectral features from two or more references even if the actual material is not a combination of the two or more reference materials. In this work, if we had measured NiS and Ni_3_S_2_ references, we could attempt a linear combination fit, but Ni in Ni@1TMoS_2_ is most certainly not simply a linear combination of these two phases or a linear combination comprising these two phases and additional phases. As a result, we respectively do not believe that the linear combination fitting would add to this study.

*12.) The EXAFS fits should be performed in a more comparative way. A fit is not good or bad on its own, it should be compared to another fit, with different parameters. This is lacking in this study. For example, the fit of the species under alkaline conditions is performed with both Ni-O and Ni-S scatterrers, it should be compared to fits where only one of the scatterrers is used, so as to see which one is best.*

**Author Response:** We appreciate the reviewer’s comments. We have considered several alternate structures and have included coordination numbers as fit parameters to account for the permutations of the model that the reviewer mentions specifically. According to this reviewer and other reviewers’ comments, as noted in the response to Question 10, we have adjusted the model for four of the Ni K-edge spectra (as-prepared and acidic spectra) to include a fixed coordination number of Ni-O of 1. If allowed to fit, the Ni-O coordination number fits to approximately 5 and gives an unreasonable first shell coordination number. After these revisions, we believe that the models utilized represent the most reasonable and best-fitting models to explain the changes observed, taking all reasonable interpretations into account. *13.) The uncertainties in table 2 should be given. Not as a general value in the caption as is done in note (b), but in brackets for each compute value. This is an important aspect of fits which also gives a sense of how good the fit is.*

**Author Response:** Per this reviewer and other reviewers’ comments, we have added the uncertainty for the fit coordination number in Table 2 and also Table 1. Uncertainties are difficult in EXAFS analysis because they are highly dependent on the fit routine. We therefore report a conservative value that represents the most reasonable level of certainty in a fit result. This is commonplace in EXAFS analysis. For distance parameters, a reasonable uncertainty as informed by the fitting results, is ,± 0.02 Å, a reasonable uncertainty in the Debye-Waller factor is ± 0.001 Å^2^, and a reasonable uncertainty in the coordination number is ± 0.5.

*14.) The migration of nickel species to form metallic nickel during the catalytic experiment is proposed on the basis of EXAFS and TEM data. Figure S10 shows a TEM image where two supposed nickel sites are next to each other, suggesting the possible formation of nickel clusters. But this image was taken from the as prepared material, which is not relevant at all, wince there is no or little metallic character in the as prepared material. Also two adjacent nickel atoms should give an average coordination number of 1 for the Ni-Ni shell observed at R’=2.0 Å, which does not seem to be the case given the intensity of the peak at the position (table 2 indicates a value of 2).*

**Author Response:** In general, most of the Ni centers appear to be separate from one another via STEM, and Figure S10 showed an example of an image where two Ni centers were found to be in proximity and surrounded by Mo centers. We agree with the reviewer that showing this image is not relevant and have removed it from the manuscript. We thank the reviewer for raising this point.

*15.) The reversibility of the metallic nickel formation would be a very interesting thing if it was clearly demonstrated. But why were data only taken ex situ? It would be much more sensible to collect data at 0V after catalysis, or at open circuit.*

**Author Response**: We agree that the data collected at an open circuit would further add to the *ex situ* data, but do not have the opportunity to collect this data. This short-term reversibility information would be interesting, but the long-term stability and reversibility from *ex situ* measurements is also quite important. Based on our post electrolysis XPS experiment and ICP-MS results, we believe that Ni@1T-MoS_2_ under acidic solution is quite stable (Figure S4, Figure S13, Table S3). However, the same catalyst under alkaline condition is not as stable. The Mo 3d, S 2p and Ni 2p peaks all shifted to higher binding energies, indicating that the catalyst is oxidized (Figure S13). With the ICP-MS results, we can see that Mo element’s concentration (μg/mL) in the solution increased after electrolysis, but no Ni leached from the catalyst (Table S3). Despite this loss of Mo, a change in the bulk structure via Raman spectroscopy (Figure S14) was not observed and local structure changes were not observed in EXAFS experiments. Changes were not observed in EXAFS likely due to the oxidized Mo species being immediately dissolved in the electrolyte in low concentration relative to the probed material. Discussion of these additional *ex situ* results has been added to the revised manuscript (Pages 18-19, lines 303-327).

Regarding the Ni sites, which were not observed to leach in alkaline conditions, we do see evidence of Ni oxidation in XPS correlating to the changes observed in EXAFS. The relevant EXAFS is discussed in Question 10 including Figure S9.

**Figure S4.** HER stability tests of NiO@1TMoS_2_ electrocatalyst. (a) the long-term durability tests in 0.5 M H_2_SO_4_ at η = 0.76 V for 1 h. (b) the long-term durability tests in 1.0 M KOH at η = 0.76 V for 1 h.

**Table S3.** The ICP-MS results of the electrolytes before and after HER stability tests of Ni@1TMoS_2_ electrocatalyst.

| Elements contents  (*μ*g/mL) | Before HER stability tests in 0.5 M H_2_SO_4_ | After HER stability tests in 0.5 M H_2_SO_4_ | Before HER stability tests in 1.0 M KOH | After HER stability tests in 1.0 M KOH |
| --- | --- | --- | --- | --- |
| Mo | <0.1 | <0.1 | <0.1 | 0.4713 |
| Ni | <0.1 | <0.1 | <0.1 | <0.1 |

**Figure S13.** XPS analysis before and after HER stability tests of Ni@1T-MoS_2_. (a-c) High-resolution XPS signals of (a) Mo3d; (b) Ni2p; (c) S2p for the Ni@1T-MoS_2_/CFP electrocatalyst.

*16.) Line 242: in the caption of figure 5, what does “the applicable results “mean?*

**Author Response:** “Applicable” means they are compared to the applicable R-space spectra, i.e. those with the most comparable structure. The Figures contain labels within them showing exactly what is compared. *17.) there is not, anywhere in the paper, any evidence for an interaction between the nickel ions and the 1T-MoS2 sheets. It is very well possible that all nickel ions detach from their substrate, migrate, and forma nanoparticles. These nanoparticles would in turn oxidise under alkaline conditions to NiO. This is actually strongly suggested by the fits under alkaline conditions (no potential or alkaline), which show N numbers >4 (corresponding to a cluster of at least 13 atoms). This value does not corresponds to the proposed structure in figure 6 (even if it is only schematic), where the N value should be ca. 1.3. These particles could then be partially reduced to metallic nickel under cathodic potential, and reoxidised to nickel oxide when brought to air. I don’t see any experimental evidence in the current version of the paper to rule out this hypothesis.*

**Author Response:** The evidence originally provided was the requirement of Mo-S paths to obtain reasonable fits on all accounts. Specifically related to the rearrangement that occurs upon immersion in alkaline media, Ni atoms become labile in the structure and form clusters associated with the 1T-MoS_2_ lattice. We have added the below figure to the SI to compare to NiO reference data to better show this structural transformation, and to also show that the species observed is not simply NiO. While XANES and XPS suggest a species electronically similar to NiO, the EXAFS show a clearly different species. EXAFS fitting corroborated this result and suggested that the dominant first shell coordinating atom of Ni becomes O after immersion in alkaline electrolyte and with a pre-catalytic potential (0V vs. RHE). We have further revised the manuscript to refer to this pre-catalytic species as NiS_x_O_y_ as discussed in Reviewer Question 10. The *ex situ* results then support that this species is re-formed after catalysis.

**Figure S9**. Comparison of NiO reference data to Ni@1T-MoS_2_ No Potential, Alkaline data. While the XANES edge (a) and first derivative (b) show similar oxidation state and features, the EXAFS oscillations (c) are significantly different. The R-space spectra were normalized at the first shell scattering feature to yield the comparison in (d) which shows that Ni in Ni@1T-MoS_2_ exhibits significantly longer first shell scattering interactions and, furthermore, shows different second shell scattering interactions. It can be interpreted that the Ni-Ni interactions in Ni@1T-MoS_2_ are much longer in the second shell, implying that the species formed is not simply NiO, but rather a species still associated with 1T-MoS_2_, i.e. NiS_x_O_y_.

*18.) The conclusion of the article is far from a sound scientific reasoning. After assuming the reversibility of their active species from nickel oxide to metallic nickel and back, the authors surmise that the nickel species can, on its own, be active at the interface of Ni@1T-MoS2. This is a very long stretch! The reversibility of the process (if proven) does not imply that the nickel at the interface is active, it is rather the opposite! It needs to change its structure (in the authors opinion migrate to form small clusters, in my opinion desorb to form nanoparticles) to be active.*

**Author Response:** We thank the reviewer for finding this confusing point in the conclusion. We have revised the statement as follows:

“These findings provided direct evidence that single-atom Ni(II) can itself perform as active species at the interface of Ni@1T-MoS_2_ in acidic conditions while it undergoes structural reconstruction in the alkaline medium to form a NiS_x_O_y_ species that reversibly forms a catalytically active Ni^0^ species under applied potential.”

*19.) Information about the synthesis and chemicals in the experimental part are too succinct. There is a long sentence from line 297 to 300, which does not make any sense to me. The method to deposit the catalysts on the carbon electrode is not properly described, which is very important. Also, the type of carbon electrode (supplier) is not indicated, which can be critical since these materials sometimes have metallic impurities. The origins of the general chemicals used are not given, which is again important because they might contain metallic impurities. (it is well know since the famous paper from Boettcher, JACS 2012, that most chemicals contain Fe impurities that can participate to catalytic activity).*

**Author Response:** We agree with the reviewer; the chemical grade and information is important for the purity of the product. Thus, we have added the chemical grade and their purity into the experimental part as follows:

All chemicals were obtained as reagent grade chemicals from Alfa Aesar® unless noted.

*Preparation of NiMo_6_ precursor*. The (NH4)_4_[NiH_6_Mo_6_O_24_]·5H_2_O (NiMo6) precursor was prepared according to a modified published procedure. (NH_4_)_6_Mo_7_O_24_·4H_2_O (denoted Mo7, 5.19 g, 4.2 mmol, 99%) was dissolved in DI water (80mL) and then heated to 100°C. Ni(NO_3_)_2_·6H_2_O (1.16 g, 4mmol, 99.99%) was dissolved in water (20mL), which was added to the above solution with stirring. The mixture was kept heating and stirring to give rise to a deep green solution. The crude product (5.4 g) was isolated with evaporation and filtration. The green targeted product (4.6 g, 79.1% yield based on Mo) was obtained by recrystallization in hot water (80 °C) two times, then dried under vacuum. Elemental analysis calcd (%) for H32N4O29NiMo6 (M=1186.60 gmol−1): H, 2.72; N, 4.72; Mo, 48.51; Found: H, 2.70; N, 4.66; Mo, 48.62. IR (KBr pellet, major absorbances, cm^−1^): 3402 (νasOH, m), 3152 (νasNH, m), 1627 (δOH, m), 1402 (δNH, s), 929 (νMo=O, vs), 876 (νMo=O, vs), 635 (νMo-O-Mo, vs), 577 (νNi-O-Mo, w).

*Preparation of Ni@1T-MoS_2_/CFP*. The as-prepared NiMo6 (50mg, 0.042mmol) precursors, thioacetamide (TAA, 80mg, 1.065 mmol, 98%) and CFP (1 × 2 cm^2^, Toray carbon paper, TGP-H-60) were mixed in 10mL H_2_O, transferred into a 20 mL Teflon autoclave, and heated at 180°C for 24 h to give rise to the corresponding Ni@1T-MoS_2_/CFP electrocatalyst. The loading amount of Ni@1T-MoS_2_ on CFP is about 1mg cm^−2^.

*All in all, this paper presents two main serious issues:
1. The lack of care in conducting the experiments presented (missing reference spectra, experimental conditions, reference molybdenum samples analysed) and in analysing them (linear fit combinations, comparative fits). Several points have been raised by the reviewer, which should have been noticed and discussed by co-authors.*

*2. There seems to be a serious mismatch between the experimental data collected and the conclusions drawn from them. The authors desperately want to show that nickel SAC are activating the base plane of MoS2, while many other hypothesis arise from the data that they present.*

**Author Response:** We have carefully and thoroughly addressed the reviewer’s questions. We thank the reviewer for the thorough review of our manuscript and the helpful suggestions to improve it. The manuscript has been revised accordingly and we hope that, with these revisions, the manuscript has improved to meet the criteria of Nature Communication.

**Reviewer comments, second version:**

Reviewer #1 (Remarks to the Author):

I cannot still recommend the paper for publication since though the authors have modified the manuscript to some extent in view of my earlier suggestions and I am now satisfied with the analysis and explanation given for the Ni K-edge data, however, the analysis of Mo K edge data and explanation of the results given in Table-1 are still very vague and according to their own admission they don't have answer to many questions themselves. Though the manuscript has important observation so far Ni K edge data are concerned, however lack of clear physical explanation of all observations does not merit it publication in a journal like Nature Communication.

Reviewer #2 (Remarks to the Author):

The authors carried out extensive revisions to the manuscript have greatly strengthened the manuscript and addressed the previous concerns satisfactorily. I have some very minor suggestions for the authors to consider. I do not need to review this manuscript again.
1. The addition of the term “NiSxOy” to denote the pre-catalytic species greatly improves comprehension of the manuscript. Figure S9 does clarify that there is a difference between this species and NiO. It seems this demonstrates the Ni-Mo path dominates the second shell, but perhaps this could be labeled explicitly. Figure S9b also appears to have a mislabeled y-axis.
2. The new references to surface reconstruction still seem somewhat lacking. A more direct example of operando EXAFS that detects a reversible surface change during electrocatalysis could be useful, such DOI: 10.1038/ncomms9625. This paper could also be suggestive of some future analysis of these Ni@1T-MoS2 catalysts that could be done with regards to their crystallinity.
3. The previously mentioned references on defects and vacancies in MoS2 (J. Am. Chem. Soc. 2016, 138, 7965, J. Am. Chem. Soc. 2016, 138, 16632) should be cited and integrated into the revised discussion.

Reviewer #3 (Remarks to the Author):

We would like to congratulate the authors for their very thorough work in addressing our comments and questions. The majority of our concerns have been answered and the quality of the paper is now suited for Nature Communications. We still think, however, that a longer format would be more appropriate for this research, so that more data and figures could be included in the main text and make the reading and understanding straightforward.

**Author rebuttal, second version:**

*Reviewer #1 (Remarks to the Author):

I cannot still recommend the paper for publication since though the authors have modified the manuscript to some extent in view of my earlier suggestions and I am now satisfied with the analysis and explanation given for the Ni K-edge data, however, the analysis of Mo K edge data and explanation of the results given in Table-1 are still very vague and according to their own admission they don't have answer to many questions themselves. Though the manuscript has important observation so far Ni K edge data are concerned, however lack of clear physical explanation of all observations does not merit it publication in a journal like Nature Communication.*

**Author Response:** We appreciate the reviewer’s comments and agree that, because major changes are not observed at the Mo K edge, the trends observed are not as definite as those observed at the Ni K edge. Therefore, as suggested by the reviewer and the editor, we have chosen to soften the claims regarding the Mo K-edge and have moved the EXAFS fitting data table for Mo K-edge (previously Table 1) into the Supplementary Information. We have shortened the discussion significantly and have moved some of the details regarding the Mo model into the Methods section. We have also combined the two paragraphs related to Mo K-edge with the Ni K-edge results/discussion under a single Results section entitled “In Situ XAS Under Acidic and Alkaline Conditions”. We have also included the following sentence to close the Mo K-edge discussions:

“Therefore, the 1T phase is shown to be the catalytically active phase in both acidic and alkaline conditions with minimal changes at the Mo center and discussion hereafter focuses on the major changes observed at the Ni K edge.”

We have chosen to keep Figure 3, which shows the XANES and EXAFS data at the Mo K-edge in the main text, because it demonstrates to the reader that there truly is little change under pre-catalytic and catalytic conditions. It further demonstrates that there is not a significant structure change, such as a bulk structure change from the 1T phase to the 2H phase, and is therefore important for the reader to reference. We believe that these changes have improved the manuscript significantly and hope that the Ni-K edge focused results section now overcomes the reviewer’s concerns.

*Reviewer #2 (Remarks to the Author):
The authors carried out extensive revisions to the manuscript have greatly strengthened the manuscript and addressed the previous concerns satisfactorily. I have some very minor suggestions for the authors to consider. I do not need to review this manuscript again.*

*1. The addition of the term “NiSxOy” to denote the pre-catalytic species greatly improves comprehension of the manuscript. Figure S9 does clarify that there is a difference between this species and NiO. It seems this demonstrates the Ni-Mo path dominates the second shell, but perhaps this could be labeled explicitly. Figure S9b also appears to have a mislabeled y-axis.*

**Author Response**: We thank the reviewer for the suggestion to label the second shell interaction in Figure S9d. We clarify that, as shown in Table 1 and discussed in the main text, the second shell interaction in the NiS_x_O_y_ species is assigned as a Ni-Ni interaction due to the presence of Ni-O-Ni moieties in the S-supported NiO clusters that we denoted NiS_x_O_y_. Because the NiO clusters in Ni@1T-MoS_2_ adopt a structure so different from bulk NiO, it is clear that the 1T-MoS_2_ lattice plays a role in structuring the pre-catalytic NiS_x_O_y_ species. For clarity, we have now labeled the second shell peaks accordingly as Ni-Ni interactions. We also thank the reviewer for noticing the mislabeling in Figure S9b, which should have the label “Derivative XA”. The updated figure is shown below.

**Supplementary Fig. 9** **Comparison of NiO reference data to Ni@1T-MoS_2_ No Potential, Alkaline data.** While the XANES edge (a) and first derivative (b) show similar oxidation state and features, the EXAFS oscillations (c) are significantly different. The R-space spectra were normalized at the first shell scattering feature to yield the comparison in (d) which shows that Ni in Ni@1T-MoS_2_ exhibits significantly longer first shell scattering interactions and, furthermore, shows different second shell scattering interactions. It can be interpreted that the Ni-Ni interactions in Ni@1T-MoS_2_ are much longer in the second shell, implying that the species formed is not simply NiO, but rather a species still associated with 1T-MoS_2_, i.e. NiS_x_O_y_.

*2. The new references to surface reconstruction still seem somewhat lacking. A more direct example of operando EXAFS that detects a reversible surface change during electrocatalysis could be useful, such DOI: 10.1038/ncomms9625. This paper could also be suggestive of some future analysis of these Ni@1T-MoS2 catalysts that could be done with regards to their crystallinity.*

**Author Response:** We thank the reviewer for the great suggestion, and have cited the reference. We also agree with the reviewer that, now that the local structure is well understood in Ni@1T-MoS_2_ under operating conditions, it could be interesting to also investigate the bulk structure in future studies.

*3. The previously mentioned references on defects and vacancies in MoS2 (J. Am. Chem. Soc. 2016, 138, 7965, J. Am. Chem. Soc. 2016, 138, 16632) should be cited and integrated into the revised discussion.*

**Author Response:** We thank the reviewer for suggesting these excellent works; We have now cited these two papers in the discussion section at lines 247-248 by incorporating a statement (underlined) into the following excerpt:

The insights gained in this study are limited by the inability to directly observe atom migration *in situ* to explain the migration of Ni atoms that are initially well-dispersed in the structure without Ni neighbours.^28^ However, surface reconstruction in 2D materials^13,46,47^ and other materials^48^ has been reported before. Relatedly, defect and vacancy sites have been shown to be important in MoS_2_-catalyzed HER.^49,50^ Furthermore, electrolyte dependent performance and electrolyte-induced dopant local structure changes have been observed in MoS_2_.^51,52^ We therefore posit that alkalinity induces Ni atom lability within the basal plane allowing the formation of Ni-O-Ni moieties, and leads to the formation of Ni^0^ under applied catalytic potential.

Because surface reconstruction is likely related to the presence of intrinsic or induced defect/vacancy sites, we believe these references fit best into this section of the work.

*Reviewer #3 (Remarks to the Author):
We would like to congratulate the authors for their very thorough work in addressing our comments and questions. The majority of our concerns have been answered and the quality of the paper is now suited for Nature Communications. We still think, however, that a longer format would be more appropriate for this research, so that more data and figures could be included in the main text and make the reading and understanding straightforward.*

**Author Response:** We thank the reviewer for the thorough job reviewing our work and believe that their comments have been a great help in the revision process.

**Reviewer comments, third version:**

**Author rebuttal, third version:**
